# Effect of Electro-Acupuncture at ST36 and SP6 on the cAMP -CREB Pathway and mRNA Expression Profile in the Brainstem of Morphine Tolerant Mice

**DOI:** 10.3389/fnins.2021.698967

**Published:** 2021-08-27

**Authors:** Qisheng Wang, Fenfen Qin, Hui Wang, Huanya Yang, Qingyang Liu, Zhonghao Li, Yongwei Jiang, Shengfeng Lu, Qian Wang, Zhigang Lu

**Affiliations:** ^1^College of Pharmacy, Nanjing University of Chinese Medicine, Nanjing, China; ^2^Key Laboratory of Acupuncture and Medicine Research of Ministry of Education, Nanjing University of Chinese Medicine, Nanjing, China; ^3^College of International Education, Nanjing University of Chinese Medicine, Nanjing, China

**Keywords:** electro-acupuncture, cAMP, PKA/ERK, CREB, morphine-induced analgesic tolerance

## Abstract

Undoubtedly, opioid drugs have been the most popular treatment for refractory pain since found, such as morphine. However, tolerance to the analgesic effects caused by repeated use is inevitable, which greatly limits the clinical application of these drugs. Nowadays, it has become the focus of the world that further development of non-opioid-based treatment along with efficient strategies to circumvent opioid tolerance are urgently needed clinically. Fortunately, electro-acupuncture (EA) provides an alternative to pharmaceutic treatment, remaining its potential mechanisms unclear although. This study was aimed to observe the effects of EA on morphine-induced tolerance in mice and discover its underlying mechanism. Tail-flick assay and hot-plate test were conducted to assess the development of tolerance to morphine-induced analgesia effect. As a result of repeated administration scheme (10 mg/kg, twice per day, for 7 days), approximately a two-fold increase was observed in the effective dose of 50% (ED50) of morphine-induced antinociceptive effect. Interestingly, by EA treatment (2/100Hz, 0.5, 1.0, and 1.5 mA, 30 min/day for 7 days) at the acupoints Zusanli (ST36) and Sanyinjiao (SP6), morphine ED50 curves was remarkably leftward shifted on day 8. In addition, the RNA sequencing strategy was used to reveal the potential mechanisms. Due to the well described relevance of cyclic adenosine monophosphate (cAMP), protein kinase A (PKA), extracellular regulated protein kinases (ERK), and cAMP response element-binding (CREB) in brainstem (BS) to analgesia tolerance, the cAMP-PKA/ERK-CREB signaling was deeply concerned in this study. Based upon Enzyme-Linked Immunosorbent Assay, the up-regulation of the cAMP level was observed, whereas reversed with EA treatment. Similarly, western blot revealed the phosphorylation levels of PKA, ERK, and CREB were up-regulated in morphine tolerant mice, whereas the EA group showed a significantly reduced expression level instead. This study observed an attenuating effect of the EA at ST36 and SP6 on morphine tolerance in mice, and suggested several potential biological targets by RNA-seq, which include the cAMP-PKA/ERK-CREB signaling pathway, strongly supporting a useful treatment for combatting the opioid epidemic, and opioid-tolerant patients.

## Introduction

Opioid analgesics, such as morphine, have been broadly used for managing moderate to severe pain management over the past decades (Caudill-Slosberg et al., 2004). However, following long-term use of opioids, there may be disturbing side effects, including analgesia tolerance, hyperalgesia, and dependence, etc., which seriously limits their clinical application and annoys the patients (DeWire et al., 2013). Morphine tolerance is probably the main cause of diminished pain control and dose escalation resulting in more widespread side effects. Consequently, in spite of pharmaceutic treatment, it is still a global urgent need to develop safe and efficient strategies to reduce opioid tolerance. As a critical part of Traditional Chinese Medicine, acupuncture is widely recommended for treating with a wide variety of diseases, which is accepted by the World Health Organization (Zhang et al., 2014). Combined with electrical stimulation, a relatively novel form of acupuncture, which is also known as electro-acupuncture (EA), has been widely used and documented in both clinical and experimental reports (Zhao, 2008; Vickers et al., 2012). Numerous evidences from trials and meta-analyses indicates that acupuncture or EA is effective for relieving pain, which may stimulate gene expression of neuropeptides and activate endogenous opioid mechanisms (Kaptchuk, 2002). Meanwhile, tolerance to electroacupuncture and its cross tolerance to morphine have been investigated since 1981. The results indicated that EA analgesia shows no cross-tolerance to morphine (Cheng et al., 1980; Han et al., 1981; Li et al., 1982). However, effects of acupuncture or electroacupuncture on morphine tolerance is remain unclear.

Morphine is an opium-derivative medication, mainly mediated by mu-opioid receptors (MOR), which are coupled to G transducer proteins and negatively coupled to adenylate cyclase (AC)(Law et al., 2000; Ream and Bruchas, 2011). Following chronic exposure to morphine, MOR-associated biased cellular signaling pathways are involved in tolerance (Colvin et al., 2019). Contrary to cAMP reduction induced by acute morphine, chronic morphine was demonstrated to induce an increase in cAMP, called cAMP overshoot or AC superactivation (vidor-Reiss et al., 1995), that activates protein kinase A (PKA) and has been demonstrated to promote morphine induced-tolerance (Smith et al., 2006; Gabra et al., 2008). Activation of cAMP-dependent kinase A results in the phosphorylation of various transcription factors, including CREB, which has been recognized as a major contributor to tolerance acquisition and proved to be responsible for the adaptive response to drug abuse (Valverde et al., 2004). It has been described that MAPKs can participate in a series of biochemical and behavioral changes induced by chronic morphine (Cui et al., 2006; Chen et al., 2008; Ma et al., 2010). Consisting of ERK1/2, JNK, and p38, the MAPKs may be candidate downstream of G-protein-coupled receptors, which transduces extracellular stimuli into intracellular transcriptional and translational responses within various pathophysiological processes (Impey et al., 1999; Widmann et al., 1999; Ji and Woolf, 2001; Ji et al., 2003). Macey et al. (2015) reported that repeated morphine administration may recruit endocytic machinery leading to the internalization and activation of endosomal signaling pathways, such as the ERK pathway, which is involved in the control of cellular responses to stress and rewarding effects (Berhow et al., 1996).

However, EA effects on experimental animal models of morphine-induced tolerance, particularly at the central nervous system level, is scarcely studied. Here, we examined the effect of EA on morphine-induced tolerance and disclosed possible mechanisms in the effect of EA stimulation.

## Materials and Methods

### Animals and Group

**FIGURE 1:**
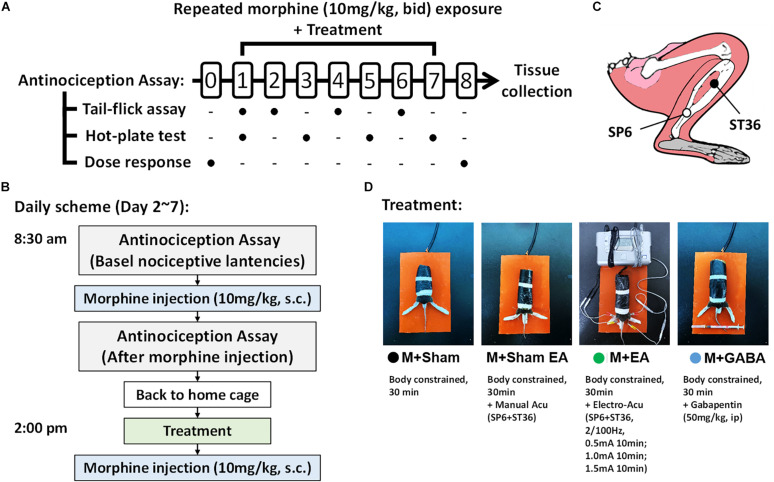
Experimental method and flow chart. **(A)** Schematic illustrating the experimental design of the analgesia tolerance paradigm. Animals were acclimated to the testing environment and investigators for at least 2 weeks. Dose–response curves were determined using a cumulative dosing scheme on day 0 and day 8. The baseline latencies of both two assays were performed at day 1. From the 1st day to the 7th day, the tail-flick test and hot-plate test will be conducted alternately to compare the effects of different treatments on the development of morphine tolerance. **(B)** The Treatment and drug administration were performed as described above in the section “Materials and Methods”. **(C)** Schematic diagram of mouse acupuncture points of SP6 and ST36. **(D)** Different treatments between groups during the 2nd day to the 7th day.

Male C57BL/6 mice (*n* = 50, 8 weeks old, SPF grade, weight 18–22 g) were obtained from the Experimental Animal Center of Nanjing University of Chinese Medicine, Nanjing, China. Animals were housed in a temperature-controlled environment on a 12 h light/dark cycle with access to food and water and a standard laboratory diet. All animals’ treatment protocols were seriously following the International ethical guidelines and the National Institute of Health Guide concerning the Care and Use of Laboratory. All experiments were approved by the committee on the Ethics of Laboratory Animal Experiments of Nanjing University of Chinese Medicine (Ethics Certificate NO. 201810A035). Each group contained ten mice. Behavioral experiments were performed from 8:00 to 18:00. The design of our animal experiments is outlined in Figure 1. To observe the effect of EA on morphine-induced tolerance, 50 mice were randomly divided into five groups: vehicle control group (V + Sham group), morphine group that received sham stimulation (M + Sham group), morphine group that received acupuncture without electro-stimulation (M + Sham EA group), morphine group that received EA stimulation (M + EA group), and morphine group that received gabapentin (M + Gaba group). The random assignment was done by the software SPSS (version 16).

### Drugs and Routes of Administration

Morphine hydrochloride and gabapentin was purchased from Jiangsu Medicine Co., Ltd. Morphine was diluted in isotonic saline and administered subcutaneously (10 mg/kg). Mice were continuously given 10mg/kg morphine twice a day (at 9 a.m. and 4 p.m.) to induce opioid tolerance, as previously described (Bohn et al., 2000; Xu et al., 2017). In this study, gabapentin was used as a positive control drug to prevent and reverse chronic opioid tolerance, which had been well established in rodents (Shimoyama et al., 1997; Gilron et al., 2003; Hamidi et al., 2014). Mice in the M + Gaba group received an injection of gabapentin diluted in saline (50 mg/kg, i.p.) immediately followed by a morphine administration every afternoon for 7 consecutive days (Stepanovic-Petrovic et al., 2008).

### EA Treatment

EA was performed similarly to that described (Liu et al., 2020; Wan et al., 2021; Wu C.X. et al., 2018). The mice remained conscious, immobilized with a home-made binding device but did not struggle violently. EA (2/100Hz, 0.5–1.5 mA) was applied to ipsilateral “Zusanli” (ST36) and “Sanyinjiao” (SP6) for 30 min, once a day for 1 week. The positioning of acupoints follows the standards of experimental acupuncture. The ST36 is located in the tibialis anterior muscle, approximately 3mm below the knee joint. SP6 is located approximately 3 mm proximal to the largest medial eminence of the posterior malleolus and between the Achilles tendon and the distal tibia. Sterile acupuncture needles (size: 0.30 × 5 mm; Beijing Zhongyan Taihe Medical Instrument Co, Ltd., Beijing, China) were inserted perpendicularly to a depth of 1 to 3 mm into the two acupoints. Han’s Acupoint Nerve Stimulator (WQ1002F, Beijing, China) provides an electro-stimulation of 30 min at a frequency of 2/100 Hz and at an intensity level of 0.5-1.0-1.5 mA to produce slight twitches in the limbs. After EA, the mice were allowed to completely free from a self-made binding instrument and further acclimatized in the cage. Mice from the EA group were treated by EA every afternoon for 7 days, whereas mice from the M + Sham group were subjected to sham EA. The animals in the M + Sham group or M + Gaba group were merely bound for 30 min in the similar self-made binding device, while other groups were treated with acupuncture or electroacupuncture. Mice in the M + Sham EA group received needle insertions at bilateral ST 36 and SP 6 acupoints without any electrical stimulation, to compare with the effects of EA on morphine tolerance. In this study, the manual acupuncture is defined as only inserting needles but without any extra handling. The main difference between MA and EA is whether the needles are electrified or not. To reduce the stress as much as possible, the acupuncture or electroacupuncture treatment was performed in a quiet, isolated room with temperature and light control between 2 pm and 4 pm. Besides, only the experimenter and assistant had free access to the room and entered the room 30 min before the treatment to eliminate potential olfactory- or auditory-induced stress. The electrical current of the EA stimulation was gradually increased from 0.5 to 1.5 mA during the treatment to minimize the discomfort of animals.

### Thermal Antinociception Assays

To assess the analgesic properties of morphine, the tail-flick assay and hot-plate test were carried out throughout this study as described with slight modification (Xu et al., 2017; Kliewer et al., 2019), both of which are highly reproducible and predictive of analgesic activity of human body (Pasternak and Pan, 2013). The investigator who performed the experiments was blinded to the treatment of each animal. Animals were acclimated to the testing environment and investigators for at least 2 weeks and randomly assigned to groups before testing. Test every 5 min, the same mouse is measured three times and then the average value was taken as the latency. The baseline latencies of both two assays were performed at day 1 (Figure 1A). Considering that either gabapentin or acupuncture (or EA) may induce certain antinociceptive effect in rodents (Stepanovic-Petrovic et al., 2008; Yu J. et al., 2019), meanwhile, in order to ensure that animals had returned to their basal nociceptive latencies, the basal latency would be measured before the challenging test.

Hot-plate test. On the 1st, 3nd, 5th, and 7th day, the paw withdrawal latencies were assessed on a hot-plate maintained at 56°C (Figure 1A). To avoid tissue damage, we used a 40-s cut off. The hot-plate test was carried out 30 min after morphine administration and expressed as percent maximum possible effect (% MPE), calculated as follows: 100 × [(response latency-basal response latency)/(40 s-basal response latency)].

Tail-flick test. Morphine antinociceptive effect was also performed with the tail-flick assay on the 2nd, 4th, and 6th day (Figure 1A), with a maximal latency of 16 sec to prevent tissue damage, Baseline latencies typically ranged around 3–4 s. The assay was carried out 30 min following a challenging dose (10 mg/kg, s.c.) of morphine administration. And data are presented as percent maximum possible effect (% MPE) according to the formula:% MPE = [(latency after drug-baseline latency)/(16- baseline latency) ^∗^100]. As shown in Figure 1A, dose–response curves were determined using a cumulative dosing scheme by the tail-flick assay on day 0 and day 8. Mice were injected at 30min intervals with 2, 4, 8, 16, 32, and 32 mg kg^–1^ morphine to yield final cumulative doses of 4, 8, 16, 32, 64, and 96 mg/kg morphine, and latencies were measured 30 min after drug administration, immediately followed by additional drug except for the last dose. ED50 values with 95% confidence intervals were determined using non-linear regression analysis (GraphPad Prism8).

### Tissue Extraction

All experimental animals were anesthetized with isoflurane (RWD, Nanjing, China). The concentration of induced anesthesia was 3–5% for tissue extraction. The animals’ brain stem was dissected quickly on a mouse Plexiglas brain mold on ice and put in liquid nitrogen for quick freezing. Then the tissues were stored at –80°C until tissue processing.

### RNA Sequencing and Bioinformatics Analysis

To investigate the underlying molecular mechanism and signaling pathways associated with EA treatment on morphine-induced tolerance, the brainstem was disserted and prepared for high-throughput sequencing. Totally, five samples per group (M + Sham and M + EA group) were sent for RNA sequencing. According to the manufacturer’s protocol, total RNA was extracted by Trizol (Takara, Japan) from the tissue. Following treatment with an rRNA remover kit (Illumina, United States), ribosomal RNAs (rRNAs) were effectively depleted to enrich pure RNAs. Use TruSeq RNA Sample Preparation Kit (Illumina) to build RNA library. Sequencing was done by Illumina Hiseq 2000 (Illumina, United States). Fold change values of >2 or < 0.05 and *P* < 0.05 were considered as differentially expressed genes. The data has been uploaded to the repository in public. to the public repository. The BioProject accession number is PRJNA721259, which allow searching in Entrez. Our SRA records is accessible with the following link: https://www.ncbi.nlm.nih.gov/sra/PRJNA721259.

### Enzyme-Linked Immunosorbent Assay (ELISA)

After homogenizing and centrifuging the mouse BS, the supernatant was used to measure the concentration of cAMP using an ELISA kit (Yu Q. et al., 2019). The expression levels of cAMP in the mouse BS were determined by the ELISA immunoassay according to the manufacturer’s protocol. All samples were assayed in duplicate. The concentration was calculated by the absorbance at 450nm. The corresponding sample concentration was calculated according to the OD value.

### Western Blot

The method of Western blotting was performed according to previous with minor modifications (Zhao et al., 2010). In brief, BS was isolated in protein lysate buffer with proteinase inhibitor phenylmethanesulfonylfluoride fluoride (PMSF) and phosphatase inhibitors. After centrifugation of the lysates (12,000 g, 10 min at 4°C), the protein concentration of each sample was determined by a BCA assay kit (Beyotime Biotechnology, Shanghai, China). Equal volumes of protein were added to each well and separated by 12% SDS-PAGE and were then transferred to a PVDF membrane. The membranes were blocked in TBST containing Tween-20 (0.1%) and BSA (5%) at room temperature for 1 h. After blocking, the membranes were incubated with primary antibodies for p-ERK (1:1000 dilution), ERK (1:1000 dilution), p-PKA (1:1000 dilution), PKA (1:1000 dilution), p-CREB (1:1000 dilution), CREB (1:1000 dilution) or β-actin (1: 1000 dilution) overnight at 4°C. After washing three times with TBST, the membranes were incubated with HRP conjugated anti-rabbit antibody (1:2000 dilution) in the TBST buffer at room temperature for 1 h. The membranes were further washed, and protein bands were identified by an enhanced chemiluminescence (ECL) reagent (Millipore, United States), imaged with a gel imaging system (Tanon, China), and quantified using Tanon image program. Data are presented as the ratio of phosphorylation proteins to total proteins.

Antibodies against ERK1/2 (#4695), phospho-ERK1/2 (#4370), PKA C (#4782s), phospho-PKA C (#5661s), CREB (#9197s), phospho-CREB (#9198s), β-actin (#4970s), and HRP-conjugated anti-rabbit secondary antibody (#7074s) were obtained from Cell Signaling Technology, United States.

### RNA Isolation and Quantitative Reverse Transcription (qRT)-PCR

RNA was extracted using trizol (Takara, Japan) following the protocols supplied by the manufacturer. Brief, the mouse BS was collected and homogenized in 1ml of trizol on ice. Total RNA (1μg) was reverse-transcribed into cDNA using PrimeScript RT kit (Toyobo, Japan). Then, real-time PCR was conducted using SYBR^®^ Green (Takara, Japan) following the manufacturer’s instructions. The primer sequences of the top 5 up-regulated and top 5 down-regulated are shown in Table 1.

**TABLE 1:** Primer sequences.

**Gene name**	**Primer type**	**Primer sequences (5′-3′)**
CHRNA6	Forwad	CCTGCACTCCGGTTTATGTCT
	Reverse	AGCGGTTGTAGTGAGCAAACA
CEBPβ	Forwad	ACCAACCGCACAT GCAGAT
	Reverse	GCAGAGGGAGAAGCAGAGAGTTT
PDE2A	Forwad	GCCGTTATCGACATTGCTGG
	Reverse	CCCCATCTAGCAGGTAGGTGTA
TM4SF4	Forwad	AAGCCACCTTTCGGATGAGG
	Reverse	CGCAGCAGTCGTTGTTCTG
KLHL38	Forwad	TGAGGAGT T ACCAGATGGGGT
	Reverse	CAGTCAGGATCTTGCTTTGTCTT
ICAM4	Forwad	AATACACTTTGCGCTGCCACGTG
	Reverse	GGCTCCAAGCGAGCATCAGTG
0IT3	Forwad	CTGATCCGTGTTGTGGTCTTC
	Reverse	GTGGCAGACGAATTGTTTTCC
CRHBP	Forwad	ATGTCACCGAACTTCAAACTCC
	Reverse	TTCTTGCACCTCTAGGTAGCG
PYY	Forwad	ACGGTCGCAATGCTGCTAAT
	Reverse	GCTGCGGGGACATCTCTTTTT
0LFM4	Forwad	CAGCCACTTTCCAATTTCACTG
	Reverse	GCTGGACATACTCCTTCACCTTA

### Statistical Analysis

All of the results are expressed as the mean ± s.e.m. Data were analyzed by GraphPad Prism software 8.0 using Student’s *t* tests, or ordinary or repeated measures one-way or two-way ANOVA, with a Bonferroni *post hoc* test, as indicated in figure captions. For dose-response curves, the best-fit line was generated following non-linear regression analysis based on the% Maximum Possible Effect (MPE) for each mouse; calculated as: MPE = [(drug induced threshold – basal threshold)/basal threshold] × 100.

## Results

### EA Ameliorated Morphine-Induced Analgesic Tolerance

**FIGURE 2:**
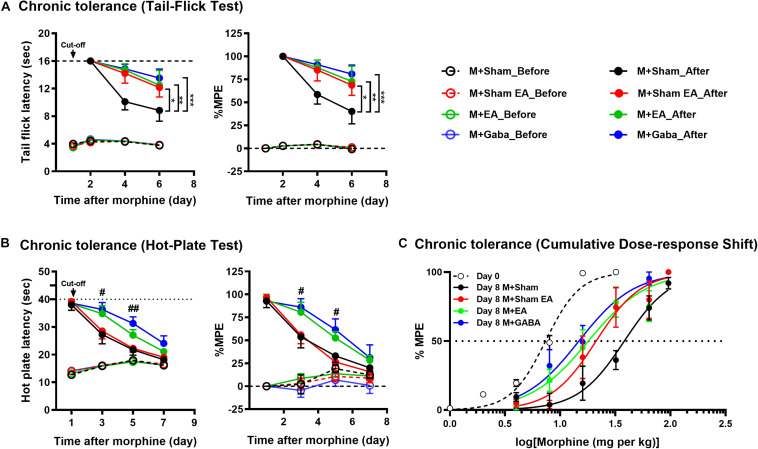
Morphine-induced chronic tolerance evaluated by **(A)** tail-flick and **(B)** hot-plate. Mice were treated daily with morphine (10 mg per kg, s.c.); antinociception was assessed 30 min after the injection on the days indicated. Symbols in graphs on left side represent mean ± SEM maximum possible effect (% MPE) or the time of latency. The data were analyzed with two-way ANOVA followed by Bonferroni *post hoc* tests. *F*(6,74) = 1.477, *F*(6,74) = 1.317. ^∗^*P* < 0.05, ^∗∗^*P* < 0.01, and ^∗∗∗^*P* < 0.001 compared to M + Sham_After. In hot-plate, day 3: left, *F*(3,26) = 3.369; right, *F*(3,26) = 5.410. day 5: left, *F*(3,25) = 3.538; right, *F*(3,25) = 7.188. ^#^*P* < 0.05, ^##^*P* < 0.01, M + GABA_After vs. M + Sham_After. **(C)** Dose-response curves were determined by tail-flick assay using a cumulative dosing scheme on day 0 to day 8. On day 1, mice were treated with 1, 2, 4, 8, and 16 mg per kg morphine (s.c.). On day 8, mice were again challenged with 4, 8, 16, 32, 64, and 96 mg per kg morphine (s.c.). ED50 (50% effective dose) values were calculated by non-linear regression analysis (GraphPad Prism). Each point in graphs represents mean ± SEM maximum possible effect (% MPE). Dotted lines indicate 50% MPE; 95% confidence intervals: day 0: 7.368 (6.859 to 7.894); day 8: M + Sham, 38.17 (30.41 to 47.10); M + Sham EA, 21.18 (15.94 to 28.44); M + EA, 18.00 (13.37 to 24.12); M + Gaba, 14.79 (11.36 to 19.13).

To confirm the effects of EA on the morphine-induced analgesic tolerance, mice were treated with morphine twice daily for 7 consecutive days and evaluated by hotplate test (An odd number of days) and tail-flick test (in the even days). The experimental process is shown in Figure 1. Mice were treated twice a day with morphine (10 mg/kg, s.c.), antinociception assessed 30 min after the first injection on the days indicated. Following 24h after morphine, significant antinociception were observed both in the tail-flick (Figure 2A) and hot-plate tests (Figure 2B) compared with the group that had received the vehicle. On day 6 or 7, antinociceptive tolerance to morphine had developed in both tests, but no morphine-induced hyperalgesia was observed in either of the tests. On day 6, M + Sham EA, M + EA, and M + Gaba group mice showed a significant analgesic effect in the assay, and the MPE value could reach approximately 75%. However, there was no significant difference between the M + Sham EA group and the M + EA group (Figure 2A, *P* > 0.05). On day 5, in the hotplate experiment, M + Gaba and M + EA mice showed a significant analgesic effect, and the MPE value could approximately reach 65%. However, there was no significant difference between the M + Sham group and the M + Sham EA group (Figure 2B, *P* > 0.05).

Then we examined the effectiveness of morphine in a dose-response scheme, comparing responsiveness on day 0 to that on day 8. Notably, after chronic daily administration, the M + EA mice and M + Gaba mice had a similar experience of a shift in their sensitivity to morphine, whereas, the M + Sham mice experienced a significant rightward shift in efficacy after continued treatment (Figure 2C).

### EA Changed Gene Expression Profiles of the Brainstem of Morphine Tolerant Mice

**FIGURE 3:**
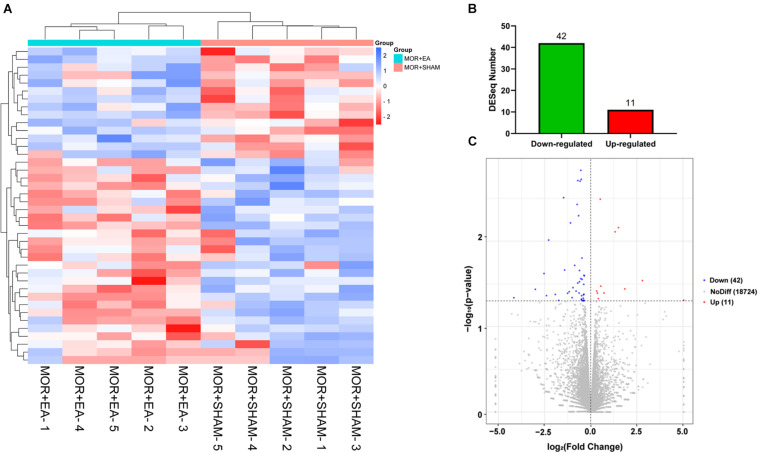
Differential expression of genes. **(A,B)** Heat map of differentially expressed genes regulated by morphine or EA. Each row represents one differentially expressed gene; each column represents one sample. The dendrogram on the left reveals the gene clustering. Green and red spectrum colors indicating downregulated and upregulated expression, respectively. **(C)** Volcano plot. *X*-axis: log_2_(fold change); *Y*-axis: log_1_0(P-value). The red points represent gene that were significantly up-regulated; the blue points represent gene that were significantly down-regulated. EA, electro-acupuncture.

We further investigated the potential molecular mechanism after confirming the effects of EA on morphine-induced mice, using the next-generation high-throughput sequencing for RNA-seq of BS. RNA was extracted from the BS, a brain area that is highly associated with tolerance, in the M + Sham, and the M + EA group. Then, unbiased deep sequencing was performed in the two groups. The general analysis results of the sequencing data are shown in Supplementary Material. In order to determine the regulation of mRNA expression, we performed an unsupervised cluster analysis of the significantly regulated genes in BS (Figure 3A). Fifty-three genes were significantly and differentially expressed in M + EA mice relative to M + Sham mice, with 11 upregulated genes and 42 downregulated genes (Figures 3B,C).

**FIGURE 4:**
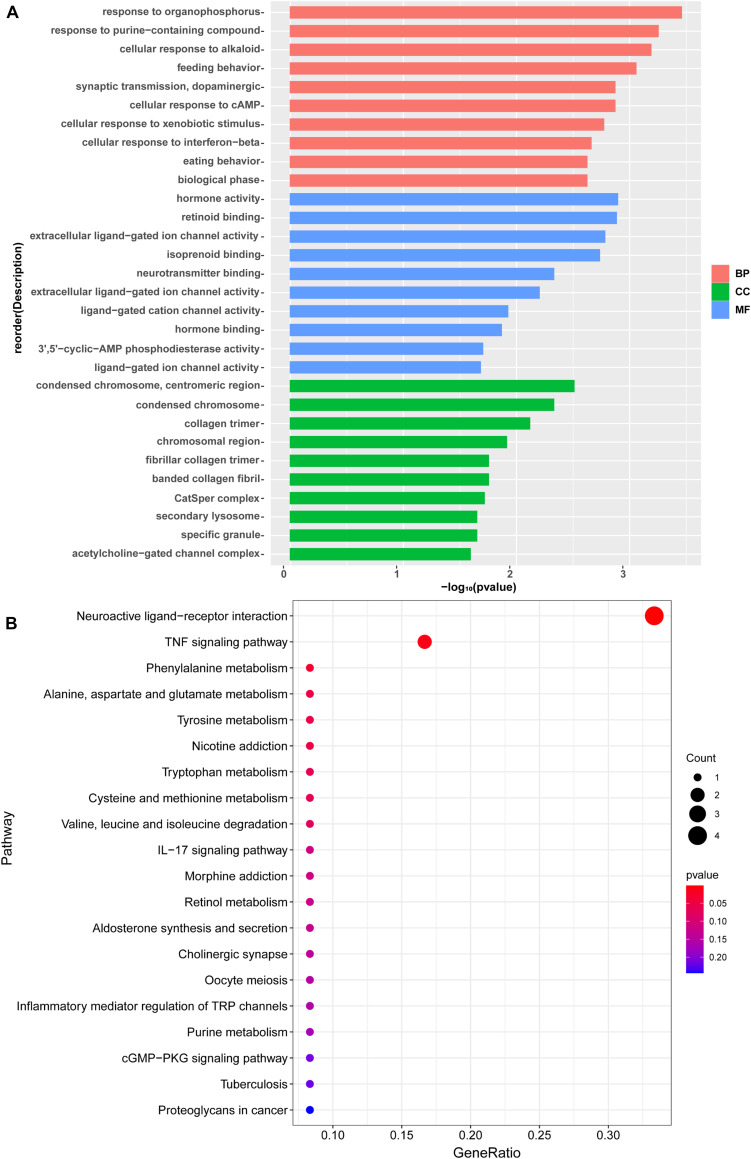
**(A)** A Gene Ontology (GO) analysis of differentially expressed gene. The enrichment score was calculated as (–log_10_[P value]). The *X*-axis is the enrichment score for the GO terms, and the *Y*-axis depicts the GO terms. Pink represent top 10 enrichment score of biological process. Green represents top 10 enrichment score of cellular component. Blue represents top 10 enrichment score of molecular function. **(B)** KEGG pathway analysis of differentially expressed with the top 20 enrichment scores. The *X*-axis represents the gene radio, and the *Y*-axis shows the name of these pathways.

**FIGURE 5:**
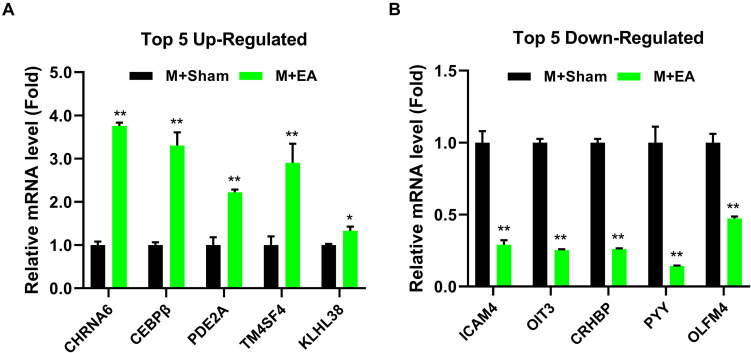
Effects of EA treatments on the expression of gene in the brainstem. **(A)** The mRNA expression level of top5 up-regulated gene in the brainstem. **(B)** The mRNA expression level of top5 down-regulated gene in the brainstem (*n* = 4). The data were analyzed with unpaired *t* test. *p < 0.05 and **p < 0.01 compared to M + Sham.

In order to show the possible cellular functions related to differentially expressed genes, we used gene ontology (GO) to enrich the analysis of differentially expressed genes across three domains, including molecular function (MF), cell composition (CC), and biological process (BP) (Figure 4A). We use all the obtained genes to perform GO function analysis to annotate and infer the function of the gene. The genes involved in cellular process, cellular response to the alkaloid, synaptic transmission, dopaminergic, cellular response to cAMP, excitatory extracellular ligand-gated ion channel activity, neurotransmitter binding, extracellular ligand-gated ion channel activity, 3′,5′-cyclic-AMP phosphodiesterase activity, secondary lysosome, and acetylcholine-gated channel complex have been reported to be related to morphine. The analysis of the Kyoto Encyclopedia of Genes and Genomes (KEGG) indicated that these genes mainly participated in metabolism, Nicotine addiction, Morphine addiction, cGMP-PKG signaling pathway, Tuberculosis, and other human diseases (Figure 4B). Among these pathways, neuroactive ligand-receptor interaction, TNF signaling pathway, Phenylalanine metabolism, Nicotine addiction, IL-17 signaling pathway, Morphine addiction, and Cholinergic synapse are related to morphine-induced tolerance. We used qPCR to verify the up-regulation and down-regulation of the top five genes, and the results were consistent with the test results (Figures 5A,B).

### Protein Expression of cAMP-p-PKA Was Regulated in Morphine Tolerant Mice With EA

**FIGURE 6:**
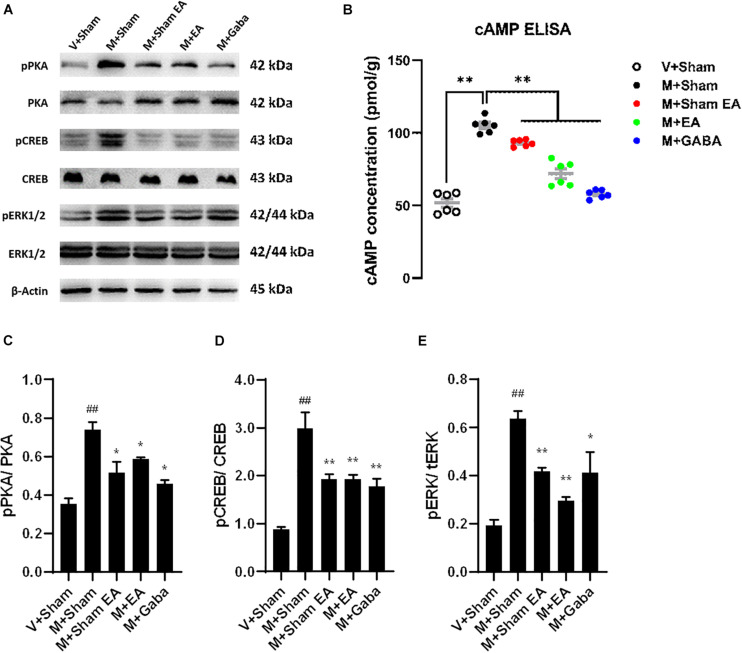
Effects of EA treatments on the protein expression in brainstem. **(A)** The brainstem tissues were lysed and analyzed by Western blotting using antibodies against PKA, phospho-PKA, ERK1/2, phospho-ERK1/2, CREB, and phospho-CREB. Representative blot was shown. GAPDH serves as loading control. **(B)** Effects of EA treatments on adenylyl cyclase activity (cAMP) after chronic morphine treatment. cAMP activity was assessed in BS after 5 days of chronic morphine treatment (10 mg/kg, s.c., per day). Data represent the mean ± SEM of experiments performed in six BS from six animals of each group assayed in triplicate in parallel experiments. **(C–E)** Graphic representation of relative expressions of pPKA/PKA, pCREB/CREB, pERK/tERK, respectively.Data are presented as the mean ± SEM (*n* = 4). The data were analyzed with one-way ANOVA followed by Bonferroni post hoc tests. *p < 0.05, ***p < 0.01, vs. M + Sham; ^##^p < 0.01, vs. V + Sham.

To verify the effects of EA on BS cAMP-p-PKA expression in morphine tolerant mice, the levels of cAMP were evaluated using ELISA. Compared with the V + Sham group, the levels of cAMP were significantly increased in morphine tolerant mice (M + Sham group) (*P* < 0.05). However, the level of cAMP was significantly decreased in M + EA group (Figure 6B, *P* < 0.05). Based upon the Western blot, the expression of p-PKA and PKA in mice BS region was also evaluated. The protein expression of p-PKA in the M + Sham group was significantly higher than V + Sham group, which was significantly reduced in the M + EA group (Figures 6A,C, *P* < 0.05).

### Protein Expression of cAMP-p-PKA Was Altered in Morphine Tolerant Mice With EA

Although the mechanism of opioid tolerance remains incompletely understood, it has been well documented that the activation of extracellular signal-regulated kinases (ERK) is prevailingly involved in signaling pathways in opioid-induced tolerance (Xu et al., 2017). Therefore, the protein expression of ERK and p-ERK was evaluated by the Western blot. Furthermore, as one of its downstream pathways, the expression of CREB and p-CREB were also evaluated. Consistent with previous observations, we found morphine-induced improve the levels of the p-ERK, whereas there were no changes in total levels of ERK in the BS. After the treatment of EA, p-ERK was decreased compared to the M + Sham group (Figures 6A,E, *P* < 0.01). Simultaneously, we found an increase in the levels of p-CREB (Figures 6A,D, *P* < 0.01), with no change in total CREB levels.

## Discussion

Long-term use of morphine will produce morphine tolerance, which leads to a decrease in analgesic effect. Alternatively, tolerance can be understood as the need to increase the dose of the drug to maintain the response. Clinically, this often is seen over days to weeks, and it is the same in animal models tolerance. Morphine, at analgesic doses, can enhance the inhibitory control of the downward pain regulation pathway derived from the brainstem. Therefore, there is an urgent need to develop strategies that can replace opioid pain management and develop strategies to reduce opioid tolerance. EA has been extensively used for treating various diseases, especially pain.

In this study, we first observed the effects of EA on morphine-induced tolerance. According to our results, after 24h of morphine injection, in the tail-flick test and the hot plate test, a significant analgesic effect was observed compared with the group receiving the vehicle. From the 2nd day of the study, a gradual decrease in the latency of tail withdrawal in the tail-flick test can be observed, indicating the development of tolerance to morphine analgesia. On the 4th day of the experiment, it can be observed that the mice in the M + Sham group showed obvious tolerance, and the MPE value was only about 50%. By the 6th day, the tolerance of the mice was more obvious, and the MPE value was lower than 50%. However, concurrent treat with EA (2/100 Hz, 0.5, 1.0, and 1.5 mA, 30 min) for 7 days effectively prevented the development of tolerance to morphine analgesia in the tail-flick test. The M + Sham EA group and the M + Gaba group were equally effective in preventing the development of morphine labor tolerance (Figure 2C).

However, the results of the hot-plate and tail-flick are different. According to our research results, on the 3rd day of morphine injection, mice in the M + Sham group and M + Sham EA group both showed obvious and similar tolerance, and the MPE value was only about 55%. At this time, the mice in the M + EA and M + Gaba groups still had a strong analgesic effect, and the MPE value could reach about 85%. By the 5th day, the tolerance of the mice was more obvious, and the MPE values of mice in the M + Sham group and M + Sham EA group were reduced to about 25%. However, although the MPE value of mice in the M + EA and M + Gaba groups was lower than that on the third day, it still reached about 55%. This also proves that the third day of continuous EA treatment can effectively prevent the development of morphine antinociceptive tolerance. The M + Gaba group was equally effective in preventing the development of morphine labor tolerance. On day 7, the analgesic effect of mice in each group was reduced to about 25%. The difference between hot plate latency and tail-flick latency may be because each method mainly reflects different levels of nociception in the central nervous system (Irwin et al., 1951; Dennis et al., 1980; Ramabadran and Bansinath, 1986). It could also be attributed to the different sensitivity of the two methods. It was assumed previously that the hot-plate predominantly reflects supraspinal response (Dennis et al., 1980). The tail-flick is considered a predominantly spinal response and may be less subject to conditioning (Irwin et al., 1951; Dennis et al., 1980). Thence, this difference may be due to the complexity of the hot-plate mechanism. The tail-flick is mainly transmitted to the central nervous system through the peripheral spinal cord, and the mechanism of the hot-plate is far more than this. Therefore, the difference in the pain threshold obtained by the hot plate method and the tail-flick method in our study may be due to the difference in the development of tolerance between the spine and the upper spine.

It is noteworthy that, after receiving chronic morphine treatment, significant analgesic tolerance was observed in mice, but no opioid-induced hyperalgesia (OIH) was observed. OIH is the increased sensitivity to pain following long-term opioid treatment, which may manifest as opioid-induced tolerance since increased sensitivity to pain would counteract the pain-relieving effects of opioids (DeWire et al., 2013). However, opioid-induced hyperalgesia is not a necessary factor for analgesic tolerance. Although both analgesic tolerance and hyperalgesia may coexist in patients who received long-term opioid treatment, it might need higher doses of opioid and longer treatment to induce OIH in uninjured model than tolerance (Liang et al., 2013; Corder et al., 2017; Doyle et al., 2020).

To reveal the potential molecular mechanism of EA-induced tolerance to morphine, RNA-seq was performed on BS. The primary reason why we chose the whole brain stem to do the mechanism analysis is that the brainstem, including the midbrain periaqueductal gray (PAG) and the rostral ventral medulla (RVM), is one of the most classical regions involved in pain transmission and modulation, knowledge of the immunohistochemistry and pharmacology of that has been well expanded since 1978 (Basbaum and Fields, 1984). Containing both high affinity opiate binding sites (Pert et al., 1975) and significant levels of endogenous opioid peptides (Hökfelt et al., 1977) makes it also to be a major research focus of morphine tolerance. In this study, we firstly decided to study the effect of electroacupuncture on the mRNA profile of the whole brainstem in morphine tolerant mice, although it may weaken the difference of gene expression in specific potential targets. Based upon the results of this study, we will further explore the target subregion and neural pathway of electroacupuncture intervention in morphine tolerance in the following research. After sequencing, we identified fifty-three genes significantly and differentially expressed in the brainstem of the two groups. The GO and KEGG analyses were used to annotate and speculate the function of the obtained genes. We found these genes were involved in cellular process, cellular response to alkaloid (Brown et al., 2012), synaptic transmission (Stockton et al., 2015), dopaminergic (Jerabek et al., 2017), cellular response to cAMP, 3′,5′-cyclic-AMP phosphodiesterase activity (Shawlutchman et al., 2002; Ligeza et al., 2008; Parlato et al., 2010), excitatory extracellular ligand-gated ion channel activity, neurotransmitter binding (Popiolek-Barczyk et al., 2018), extracellular ligand-gated ion channel activity (Sorrell and Hauser, 2014), which have been reported to be associated with morphine. Meanwhile, we also found these genes mainly participated in seven categories of pathways, including TNF signaling pathway, IL-17 signaling pathway, neuroactive ligand-receptor interaction, Phenylalanine metabolism, Nicotine addiction, Morphine addiction, and Cholinergic synapse. Among these pathways, the TNF signaling pathway (Wu X.P. et al., 2018), IL-17 signaling pathway (Banerjee et al., 2015), neuroactive ligand-receptor interaction, Nicotine addiction, Morphine addiction have been previously reported to be in connection with morphine.

Previous reports showed: cAMP response element binding protein (of CREB) is a nuclear protein, the promoter regulating the transcription of a gene having a cAMP response element (Johannessen et al., 2004). Kinases including ERK1/2 and PKA can activate CREB, thereby inducing its transcriptional activity, and CREB activation has been detected under pain conditions of different origins (Descalzi et al., 2012; Galeotti and Ghelardini, 2013). Previous studies have shown that the PKA contributes to the expression of morphine tolerance, and the behavioral and physiological effects of morphine in morphine induced tolerant mice could be reinstated by the kinase inhibitors interacting solely with the morphine released from the pellet (Smith et al., 2006).

In the following research, ELISA and Western blot were used to study the effects of EA on the expression of cAMP-PKA/ERK-CREB pathway in the brainstem of morphine tolerant mice. The results showed that the levels of cAMP were increased in the M + Sham group than that in the con group, whereas decreased in the M + EA group, compared with the M + Sham. Based upon no variation of PKA, ERK, and CREB detected within all groups, the phosphorylation of ERK and CREB was increased in the M + Sham group compared with the V + Sham group, which were alleviated in the M + EA group, demonstrated a potential cellular signaling cascade involved with the effect of EA. However, the phosphorylation in the downstream pathways could also be regulated by other pathways. For example, p-ERK1/2 was regulated by G protein and/or β-arrestin2 through mu-opioid receptors. In the next step, we will conduct more in-depth research to explore more targets for EA to treat morphine-induced tolerance.

**FIGURE 7:**
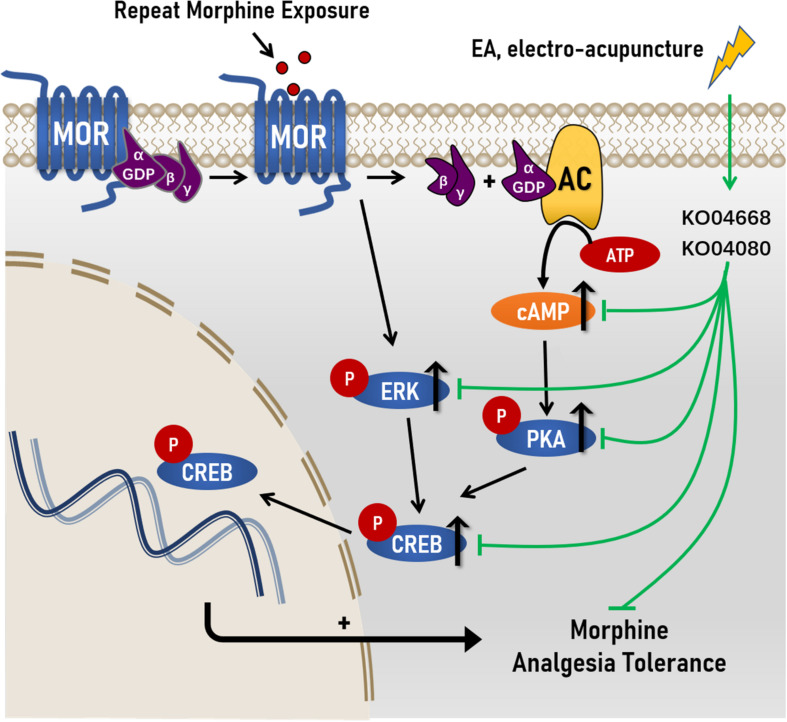
Proposed mechanisms of action by which EA ameliorated morphine-induced tolerance in mice. Chronic morphine increased the levels of cAMP, and the levels of phosphorylation levels of protein kinase A (PKA), extracellular regulated protein kinases (ERK) and cAMP response element-binding (CREB) in morphine-induced tolerance. After the treatment of Electro-Acupuncture, the increased of cAMP, p-PKA C, p-ERK, and p-CREB were significantly decreased in morphine-induce tolerance. Simultaneously, RNA sequencing reval the underlying molecular targets of EA treatment on morphine-induced tolerance maybe KO04080 (Neuroactive ligand-receptor interaction) and KO04668 (TNF signaling pathway).

Taken together, these data suggested that the modulation of cAMP-PKA/ERK-CREB signaling pathway and the possible cellular functions related to differentially expressed genes may be potentially associated with the effect of EA at ST36 and SP6 on the development of antinociceptive tolerance to morphine. This study also provided a new potential treatment method for clinical intervention of morphine tolerance that EA treatment is supported to be used in patients who need to use long-term opioids, to alleviate the development of tolerance and dose escalation (Figure 7).

## Data Availability Statement

The datasets presented in this study can be found in online repositories. The names of the repository/repositories and accession number(s) can be found below: https://www.ncbi.nlm.nih.gov/sra/PRJNA721259.

## Ethics Statement

All experiment was approved by the Committee on the Ethics of Laboratory Animal Experiments of Nanjing University of Chinese Medicine.

## Author Contributions

QsW and FQ participated in designing experiments, carried out the experiments in the study, and prepared the first draft of the manuscript. ZL and QaW conceived of the study and participated in its design and polished the language. HY, ZL, QL, HW, and YJ performed a part of experiments and bioinformatics analysis. SL participated in experimental design and consulted on the study. All authors approved the final manuscript.

## Conflict of Interest

The authors declare that the research was conducted in the absence of any commercial or financial relationships that could be construed as a potential conflict of interest.

## Publisher’s Note

All claims expressed in this article are solely those of the authors and do not necessarily represent those of their affiliated organizations, or those of the publisher, the editors and the reviewers. Any product that may be evaluated in this article, or claim that may be made by its manufacturer, is not guaranteed or endorsed by the publisher.

## References

[B1] Caudill-Slosberg M. A. Schwartz L. M. Woloshin S. J. P. (2004). Office visits and analgesic prescriptions for musculoskeletal pain in US: 1980 vs. 2000. *Pain* 109 514–519. 10.1016/s0304-3959(04)00130-7 15157714

[B2] DeWire S. M. Yamashita D. S. Rominger D. H. Liu G. Cowan C. L. Graczyk T. M. (2013). A G protein-biased ligand at the μ-opioid receptor is potently analgesic with reduced gastrointestinal and respiratory dysfunction compared with morphine. *J. Pharmacol. Exp. Ther.* 344 708–717. 10.1124/jpet.112.201616 23300227

[B3] Zhang R. Lao L. Ren K. Berman B. M. (2014). Mechanisms of acupuncture-electroacupuncture on persistent pain. *Anesthesiology* 120 482–503. 10.1097/aln.0000000000000101 24322588 PMC3947586

[B4] Vickers A. J. Cronin A. M. Maschino A. C. Lewith G. MacPherson H. Foster N. E. (2012). Acupuncture for chronic pain: individual patient data meta-analysis. *J. Arch. Intern. Med.* 55 24–25. 10.1001/archinternmed.2012.3654 PMC3658605 22965186

[B5] Zhao Z. Q. (2008). Neural mechanism underlying acupuncture analgesia. *Prog. Neurobiol.* 85 355–375. 10.1016/j.pneurobio.2008.05.004 18582529

[B6] Kaptchuk T. J. (2002). Acupuncture: theory, efficacy, and practice. *Ann. Intern. Med.* 136 374–383. 11874310 10.7326/0003-4819-136-5-200203050-00010

[B7] Li S. J. Tang J. Han J. S. (1982). The implication of central serotonin in electro-acupuncture tolerance in the rat. *Sci. Sin. B* 25 620–629. 6180474

[B8] Cheng R. S. Pomeranz B. Yu G. (1980). Electroacupuncture treatment of morphine-dependent mice reduces signs of withdrawal, without showing cross-tolerance. *Eur. J. Pharmacol.* 68 477–481. 10.1016/0014-2999(80)90423-9 7193585

[B9] Han J. S. Li S. J. Tang J. (1981). Tolerance to electroacupuncture and its cross tolerance to morphine. *Neuropharmacology* 20 593–596. 10.1016/0028-3908(81)90213-6 7195480

[B10] Law P. Y. Wong Y. H. Loh H. H. (2000). Molecular mechanisms and regulation of opioid receptor signaling. *Annu. Rev. Pharmacol. Toxicol.* 40 389–430. 10.1146/annurev.pharmtox.40.1.389 10836142

[B11] Ream A. H. Bruchas M. R. (2011). Molecular mechanisms of opioid receptor-dependent signaling and behavior. *Anesthesiology* 115 1363–1381. 10.1097/aln.0b013e318238bba6 22020140 PMC3698859

[B12] Colvin L. A. Bull F. Hales T. G. (2019). Perioperative opioid analgesia-when is enough too much? A review of opioid-induced tolerance and hyperalgesia. *Lancet* 393 1558–1568. 10.1016/s0140-6736(19)30430-1 30983591

[B13] vidor-Reiss T. A. Bayewitch M. Levy R. Matus-Leibovitch N. Nevo I. Vogel Z. (1995). Adenylylcyclase supersensitization in μ-opioid receptor-transfected chinese hamster ovary cells following chronic opioid treatment. *J. Biol. Chem.* 270:29732. 10.1074/jbc.270.50.29732 8530363

[B14] Gabra B. H. Bailey C. P. Kelly E. Smith F. L. Henderson G. Dewey W. L. (2008). Pre-treatment with a PKC or PKA inhibitor prevents the development of morphine tolerance but not physical dependence in mice. *Brain Res.* 1217 70–77. 10.1016/j.brainres.2008.04.036 18501877 PMC3773693

[B15] Smith F. L. Javed R. R. Smith P. A. Dewey W. L. Gabra B. H. (2006). PKC and PKA inhibitors reinstate morphine-induced behaviors in morphine tolerant mice. *Pharmacol. Res.* 54 474–480. 10.1016/j.phrs.2006.09.007 17056270

[B16] Valverde T. Mantamadiotis M. Torrecilla L. Ugedo J. Pineda S. Bleckmann P. (2004). Modulation of anxiety-like behavior and morphine dependence in CREB-deficient mice. *Neuropsychopharmacology* 29 1122–1133. 10.1038/sj.npp.1300416 15029152

[B17] Ma W. Zheng W. H. Powell K. Jhamandas K. Quirion R. (2010). Chronic morphine exposure increases the phosphorylation of MAP kinases and the transcription factor CREB in dorsal root ganglion neurons: an in vitro and in vivo study. *Eur. J. Neurosci.* 14 1091–1104. 10.1046/j.0953-816x.2001.01731.x 11683901

[B18] Cui Y. Chen Y. Zhi J.-L. Guo R.-X. Feng J.-Q. Chen P.-X. (2006). Activation of p38 mitogen-activated protein kinase in spinal microglia mediates morphine antinociceptive tolerance. *Brain Res.* 1069 235–243. 10.1016/j.brainres.2005.11.066 16403466

[B19] Chen Y. Geis C. Sommer C. (2008). Activation of TRPV1 contributes to morphine tolerance: involvement of the mitogen-activated protein kinase signaling pathway. *J. Neurosci.* 28 5836–5845. 10.1523/jneurosci.4170-07.2008 18509045 PMC6670790

[B20] Ji R. R. Kohno T. Moore K. A. Woolf C. J. (2003). Central sensitization and LTP: do pain and memory share similar mechanisms? *Trends Neurosci.* 26 696–705. 10.1016/j.tins.2003.09.017 14624855

[B21] Impey S. Obrietan K. Storm D. R. (1999). Making new connections: role of ERK/MAP kinase signaling in neuronal plasticity. *Neuron* 23 11–14. 10402188 10.1016/s0896-6273(00)80747-3

[B22] Widmann C. Gibson S. Jarpe M. B. Johnson G. L. (1999). Mitogen-activated protein kinase: conservation of a three-kinase module from yeast to human. *Physiol. Rev.* 79 143–180. 10.1152/physrev.1999.79.1.143 9922370

[B23] Ji R. R. Woolf C. J. (2001). Neuronal plasticity and signal transduction in nociceptive neurons: implications for the initiation and maintenance of pathological pain. *Neurobiol. Dis.* 8 1–10. 10.1006/nbdi.2000.0360 11162235

[B24] Macey T. A. Bobeck E. N. Suchland K. L. Morgan M. M. Ingram S. L. (2015). Change in functional selectivity of morphine with the development of antinociceptive tolerance. *Br. J. Pharmacol.* 172 549–561. 10.1111/bph.12703 24666417 PMC4292967

[B25] Berhow M. T. Hiroi N. Nestler E. J. (1996). Regulation of ERK (extracellular signal regulated kinase), part of the neurotrophin signal transduction cascade, in the rat mesolimbic dopamine system by chronic exposure to morphine or cocaine. *J. Neurosci.* 16 4707–4715. 10.1523/jneurosci.16-15-04707.1996 8764658 PMC6579030

[B26] Bohn L. M. Gainetdinov R. R. Lin F. T. Lefkowitz R. J. Caron M. G. (2000). Mu-opioid receptor desensitization by beta-arrestin-2 determines morphine tolerance but not dependence. *Nature* 408 720–723. 10.1038/35047086 11130073

[B27] Xu J. Lu Z. Narayan A. Le Rouzic V. P. Xu M. Hunkele A. (2017). Alternatively spliced mu opioid receptor C termini impact the diverse actions of morphine. *J. Clin. Invest.* 127 1561–1573. 10.1172/jci88760 28319053 PMC5373896

[B28] Gilron I. Biederman J. Jhamandas K. Hong M. (2003). Gabapentin blocks and reverses antinociceptive morphine tolerance in the rat paw-pressure and tail-flick tests. *Anesthesiology* 98 1288–1292. 10.1097/00000542-200305000-00037 12717156

[B29] Hamidi G. A. Jafari-Sabet M. Abed A. Mesdaghinia A. Mahlooji M. Reza Banafshe H. (2014). Gabapentin enhances anti-nociceptive effects of morphine on heat, cold, and mechanical hyperalgesia in a rat model of neuropathic pain. *Iran. J. Basic Med. Sci.* 17 753–759. 25729543 PMC4340982

[B30] Shimoyama M. Shimoyama N. Inturrisi C. E. Elliott K. J. (1997). Gabapentin enhances the antinociceptive effects of spinal morphine in the rat tail-flick test. *Pain* 72 375–382. 10.1016/s0304-3959(97)00065-1 9313278

[B31] Stepanovic-Petrovic R. M. Tomic M. A. Vuckovic S. M. Paranos S. Ugresic N. D. Prostran M. S. (2008). The antinociceptive effects of anticonvulsants in a mouse visceral pain model. *Anesth. Analg.* 106 1897–1903. 10.1213/ane.0b013e318172b993 18499629

[B32] Liu S. Wang Z.-F. Su Y.-S. Ray R. S. Jing X.-H. Wang Y.-Q. (2020). Somatotopic organization and intensity dependence in driving distinct NPY-expressing sympathetic pathways by electroacupuncture. *Neuron* 108 436–450. 10.1016/j.neuron.2020.07.015 32791039 PMC7666081

[B33] Wan C. Xu Y. Cen B. Xia Y. Yao L. Zheng Y. (2021). Neuregulin1-ErbB4 signaling in spinal cord participates in electroacupuncture analgesia in inflammatory pain. *Front. Neurosci.* 15:636348. 10.3389/fnins.2021.636348 33584196 PMC7875897

[B34] Wu C.-X. Feng Y.-H. Yang L. Zhan Z.-L. Xu X.-H. Hu X.-Y. (2018). Electroacupuncture exerts neuroprotective effects on ischemia/reperfusion injury in JNK knockout mice: the underlying mechanism. *Neural Regen. Res.* 13 1594–1601. 10.4103/1673-5374.235294 30127120 PMC6126120

[B35] Kliewer A. Schmiedel F. Sianati S. Bailey A. Bateman J. T. Levitt E. S. (2019). Phosphorylation-deficient G-protein-biased μ-opioid receptors improve analgesia and diminish tolerance but worsen opioid side effects. *Nat. Commun.* 10:367. 10.1038/s41467-018-08162-1 PMC6341117 30664663

[B36] Pasternak G. W. Pan Y.-X. (2013). Mu opioids and their receptors: evolution of a concept. *Pharmacol. Rev.* 65 1257–1317. 10.1124/pr.112.007138 24076545 PMC3799236

[B37] Yu J. Wang D.-S. Bonin R. P. Penna A. Alavian-Ghavanini A. Zurek A. A. (2019). Gabapentin increases expression of δ subunit-containing GABA receptors. *EBioMedicine* 42 203–213. 10.1016/j.ebiom.2019.03.008 30878595 PMC6491385

[B38] Yu Q. Shuai H. Ahooghalandari P. Gylfe E. Tengholm A. (2019). Glucose controls glucagon secretion by directly modulating cAMP in alpha cells. *Diabetologia* 62 1212–1224. 10.1007/s00125-019-4857-6 30953108 PMC6560012

[B39] Zhao Y. Luo D. Ning Z. Rong J. Lao L. (2010). Electro-acupuncture ameliorated MPTP-induced parkinsonism in mice via TRKB neurotrophic signaling. *Front. Neurosci.* 13:496. 10.3389/fnins.2019.00496 31156376 PMC6528026

[B40] Irwin S. Houde R. W. Bennett D. R. Hendershot L. C. Seevers M. H. (1951). The effects of morphine, methadone and meperidine on some reflex responses of spinal animals to nociceptive stimulation. *J. Pharmacol. Exp. Ther.* 101 132–143. 14814606

[B41] Dennis S. G. Melzack R. Gutman S. Boucher F. (1980). Pain modulation by adrenergic agents and morphine as measured by three pain tests. *Life Sci.* 26 1247–1259. 10.1016/0024-3205(80)90070-3 7392798

[B42] Ramabadran K. Bansinath M. (1986). A critical analysis of the experimental evaluation of nociceptive reactions in animals. *Pharm. Res.* 3 263–270. 24271708 10.1023/A:1016355200944

[B43] Liang D.-Y. Li X. Clark J. D. (2013). Epigenetic regulation of opioid-induced hyperalgesia, dependence, and tolerance in mice. *J. Pain* 14 36–47. 10.1016/j.jpain.2012.10.005 23273833 PMC3539745

[B44] Doyle T. M. Largent-Milnes T. M. Chen Z. Staikopoulos V. Esposito E. Dalgarno R. (2020). Chronic morphine-induced changes in signaling at the a adenosine receptor contribute to morphine-induced hyperalgesia, tolerance, and withdrawal. *J. Pharmacol. Exp. Ther.* 374 331–341. 10.1124/jpet.120.000004 32434943 PMC7372916

[B45] Corder G. Tawfik V. L. Wang D. Sypek E. I. Low S. A. Dickinson J. R. (2017). Loss of μ opioid receptor signaling in nociceptors, but not microglia, abrogates morphine tolerance without disrupting analgesia. *Nat. Med.* 23 164–173. 10.1038/nm.4262 28092666 PMC5296291

[B46] Basbaum A. I. Fields H. L. (1984). Endogenous pain control systems: brainstem spinal pathways and endorphin circuitry. *Annu. Rev. Neurosci.* 7 309–338. 10.1146/annurev.ne.07.030184.001521 6143527

[B47] Pert C. B. Kuhar M. J. Snyder S. H. (1975). Autoradiograhic localization of the opiate receptor in rat brain. *Life Sci.* 16 1849–1853. 10.1016/0024-3205(75)90289-1 1152615

[B48] Hökfelt T. Ljungdahl A. Terenius L. Elde R. Nilsson G. (1977). Immunohistochemical analysis of peptide pathways possibly related to pain and analgesia: enkephalin and substance. *Proc. Natl Acad. Sci. U.S.A.* 74 3081–3085. 10.1073/pnas.74.7.3081 331326 PMC431417

[B49] Brown J. N. Ortiz G. M. Angel T. E. Jacobs J. M. Gritsenko M. Chan E. Y. (2012). Morphine produces immunosuppressive effects in nonhuman primates at the proteomic and cellular levels. *Mol. Cell. Proteomics* 11 605–618. 10.1074/mcp.m111.016121 22580588 PMC3434775

[B50] Stockton S. D. Jr. Gomes I. Liu T. Moraje C. Hipólito L. Jones M. R. (2015). Morphine regulated synaptic networks revealed by integrated proteomics and network analysis. *J. Mol. Cell. Proteomics* 14 2564. 10.1074/mcp.m115.047977 26149443 PMC4597137

[B51] Jerabek P. Havlickova T. Puskina N. Charalambous C. Lapka M. Kacer P. (2017). Ghrelin receptor antagonism of morphine-induced conditioned place preference and behavioral and accumbens dopaminergic sensitization in rats. *Neurochem. Int.* 110 101–113. 10.1016/j.neuint.2017.09.013 28958601

[B52] Parlato R. Cruz H. Otto C. Murtra P. Parkitna J. R. Martin M. (2010). Effects of the cell type-specific ablation of the cAMP-responsive transcription factor in noradrenergic neurons on locus coeruleus firing and withdrawal behavior after chronic exposure to morphine. *J. Neurochem.* 115 563–573. 10.1111/j.1471-4159.2010.06709.x 20367754

[B53] Shawlutchman T. Z. Barrot M. Wallace T. Gilden L. Zachariou V. Impey S. (2002). Regional and cellular mapping of cAMP response element-mediated transcription during naltrexone-precipitated morphine withdrawal. *J. Neurosci.* 22:3663. 10.1523/jneurosci.22-09-03663.2002 11978842 PMC6758390

[B54] Ligeza A. Wawrzczak-Bargiela A. Kaminska D. Korostynski M. Przewlocki R. (2008). Regulation of ERK1/2 phosphorylation by acute and chronic morphine – implications for the role of cAMP-responsive element binding factor (CREB)-dependent and Ets-like protein-1 (Elk-1)-dependent transcription; small interfering RNA-based strategy. *FEBS J.* 275 3836–3849. 10.1111/j.1742-4658.2008.06531.x 18616461

[B55] Popiolek-Barczyk K. Łażewska D. Latacz G. Olejarz A. Makuch W. Stark H. (2018). Antinociceptive effects of novel histamine H 3 3 and H 4 4 receptor antagonists and their influence on morphine analgesia of neuropathic pain in the mouse. *Br. J. Pharmacol.* 175 2897–2910. 10.1111/bph.14185 29486058 PMC6016676

[B56] Sorrell M. E. Hauser K. F. (2014). Ligand-gated purinergic receptors regulate HIV-1 tat and morphine related neurotoxicity in primary mouse striatal neuron-glia co-cultures. *J. Neuroimmune Pharmacol.* 9 233–244. 10.1007/s11481-013-9507-z 24158495 PMC3959217

[B57] Wu X. P. She R.-X. Yang Y.-P. Xing Z.-M. Chen H.-W. Zhang Y.-W. (2018). MicroRNA-365 alleviates morphine analgesic tolerance via the inactivation of the ERK/CREB signaling pathway by negatively targeting β-arrestin2. *J. Biomed. Sci.* 25:10. 10.1186/s12929-018-0405-9 PMC5802062 29415719

[B58] Banerjee S. Ninkovic J. Meng J. Sharma U. Ma J. Charboneau R. (2015). Morphine compromises bronchial epithelial TLR2/IL17R signaling crosstalk, necessary for lung IL17 homeostasis. *Sci. Rep.* 5:11384. 10.1038/srep11384 PMC4466887 26072707

[B59] Johannessen M. Delghandi M. P. Moens U. (2004). What turns CREB on? *Cell Signal* 16 1211–1227. 10.1016/j.cellsig.2004.05.001 15337521

[B60] Descalzi G. Fukushima H. Suzuki A. Kida S. Zhuo M. (2012). Genetic enhancement of neuropathic and inflammatory pain by forebrain upregulation of CREB-mediated transcription. *J. Mol. Pain* 8:90. 10.1186/1744-8069-8-90 PMC3545978 23272977

[B61] Galeotti N. Ghelardini C. (2013). Reversal of NO-induced nociceptive hypersensitivity by St. John’s wort and hypericin: NF-κB, CREB and STAT1 as molecular targets. *Psychopharmacology* 227 149–163. 10.1007/s00213-012-2950-3 23254377

