# Supplementary figures and images for: Effect of Electro-Acupuncture at ST36 and SP6 on the cAMP -CREB Pathway and mRNA Expression Profile in the Brainstem of Morphine Tolerant Mice
Source: Front Neurosci. 2021 Aug 27;15:698967. doi: 10.3389/fnins.2021.698967 (PMC8431970; doi:10.3389/fnins.2021.698967)

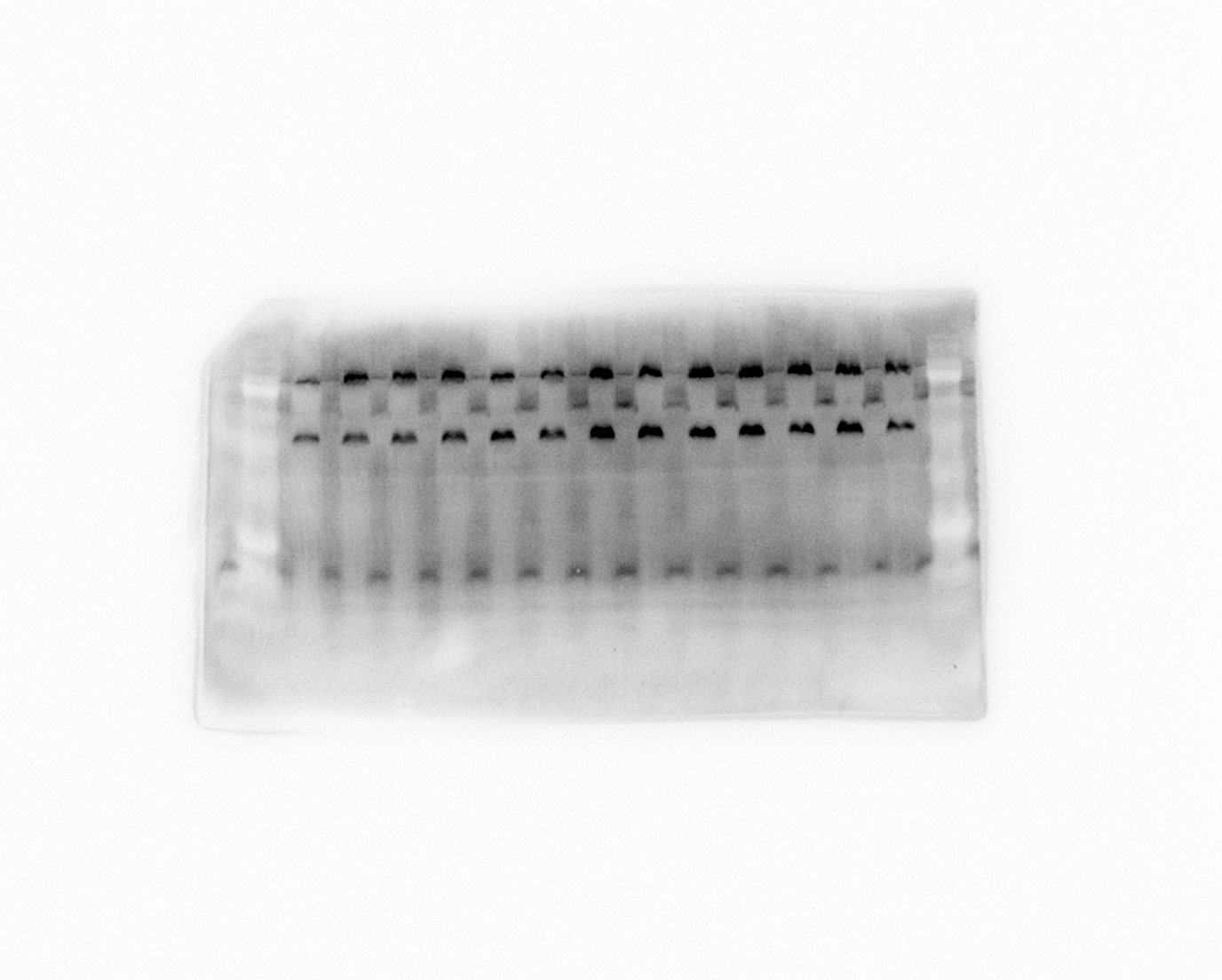

Supplement: Supplementary file 1 [file Data_Sheet_1.ZIP › Supplementary materials 1/WB/creb.tif]

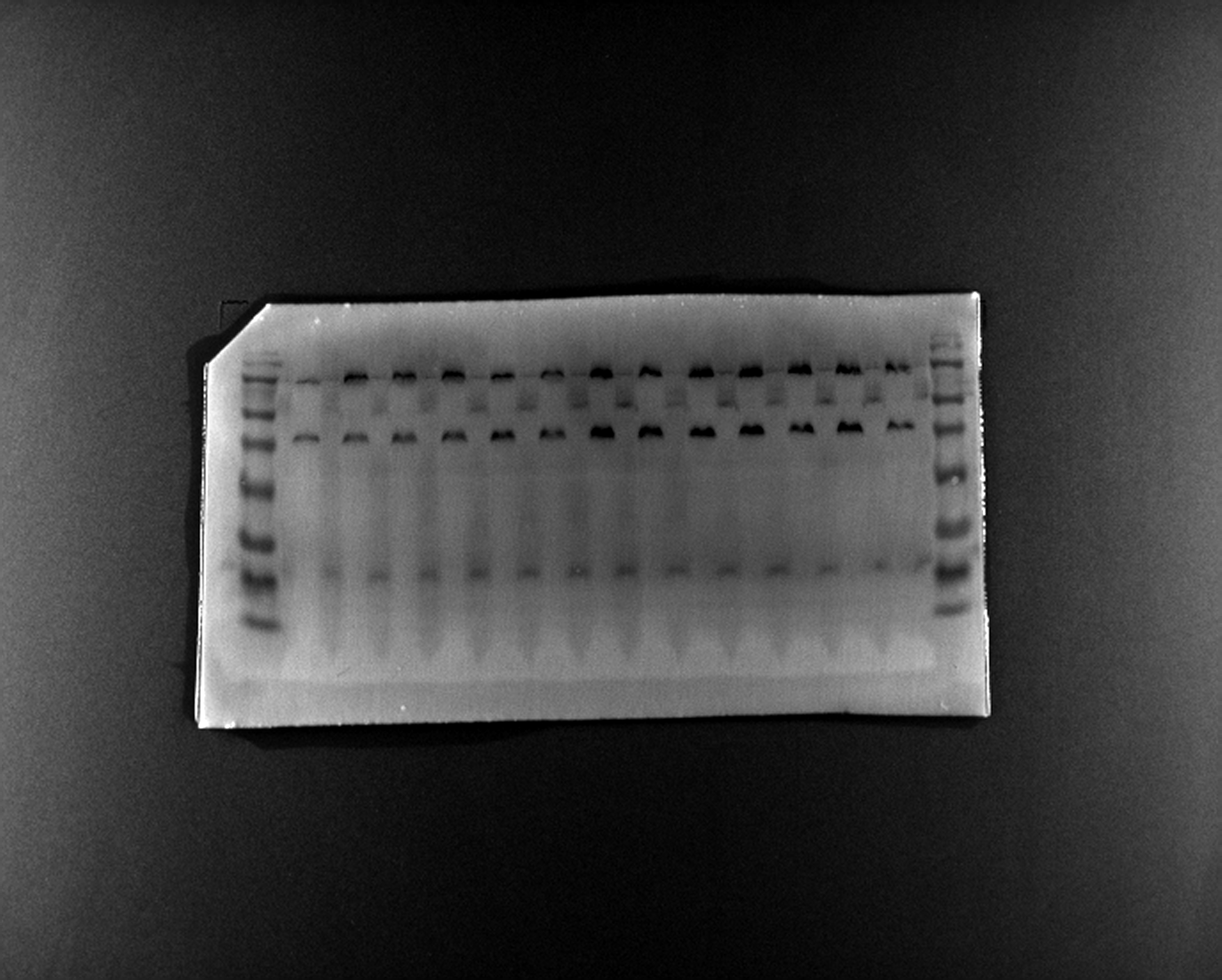

Supplement: Supplementary file 1 [file Data_Sheet_1.ZIP › Supplementary materials 1/WB/creb-merge.tif]

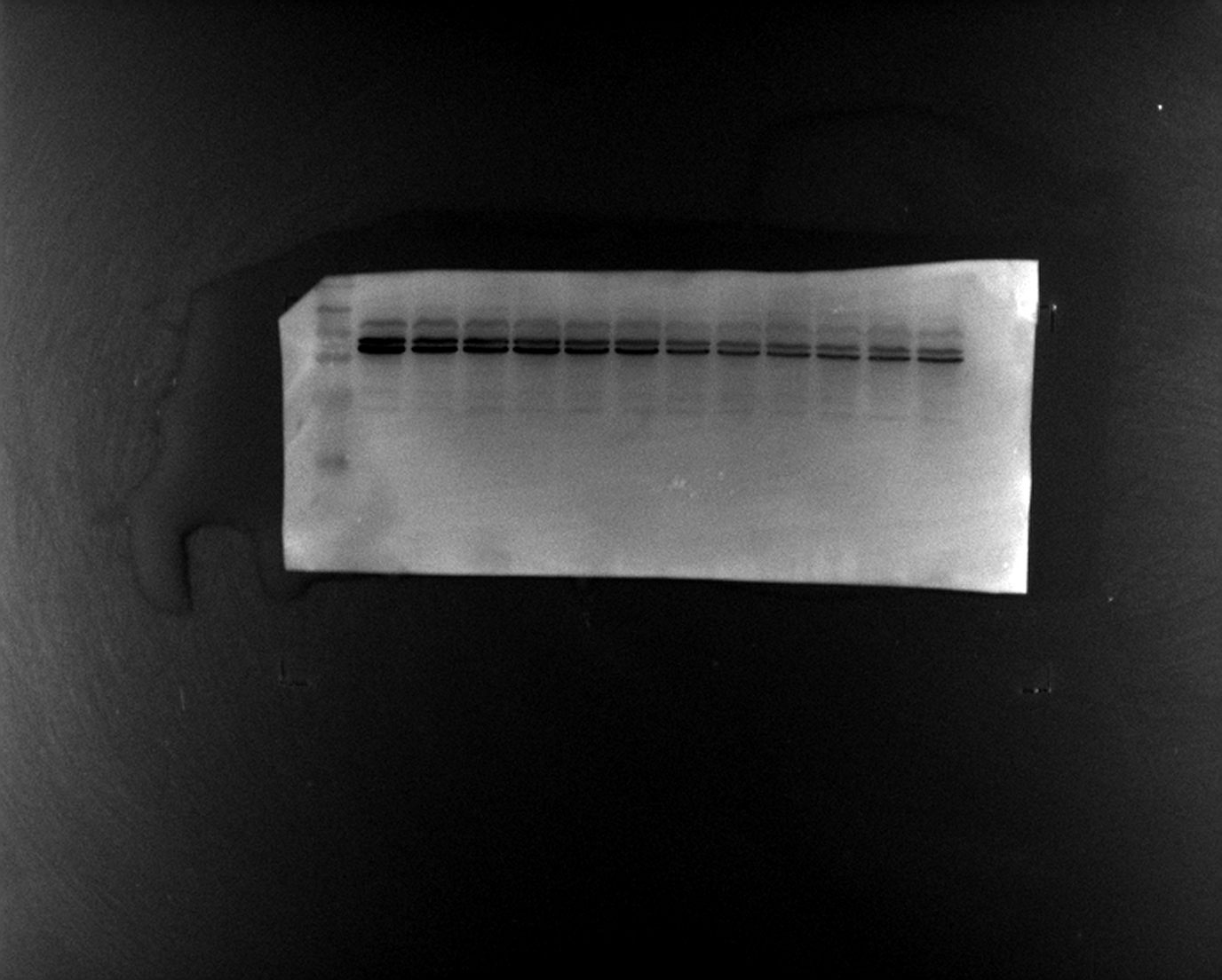

Supplement: Supplementary file 1 [file Data_Sheet_1.ZIP › Supplementary materials 1/WB/ERK merge.tif]

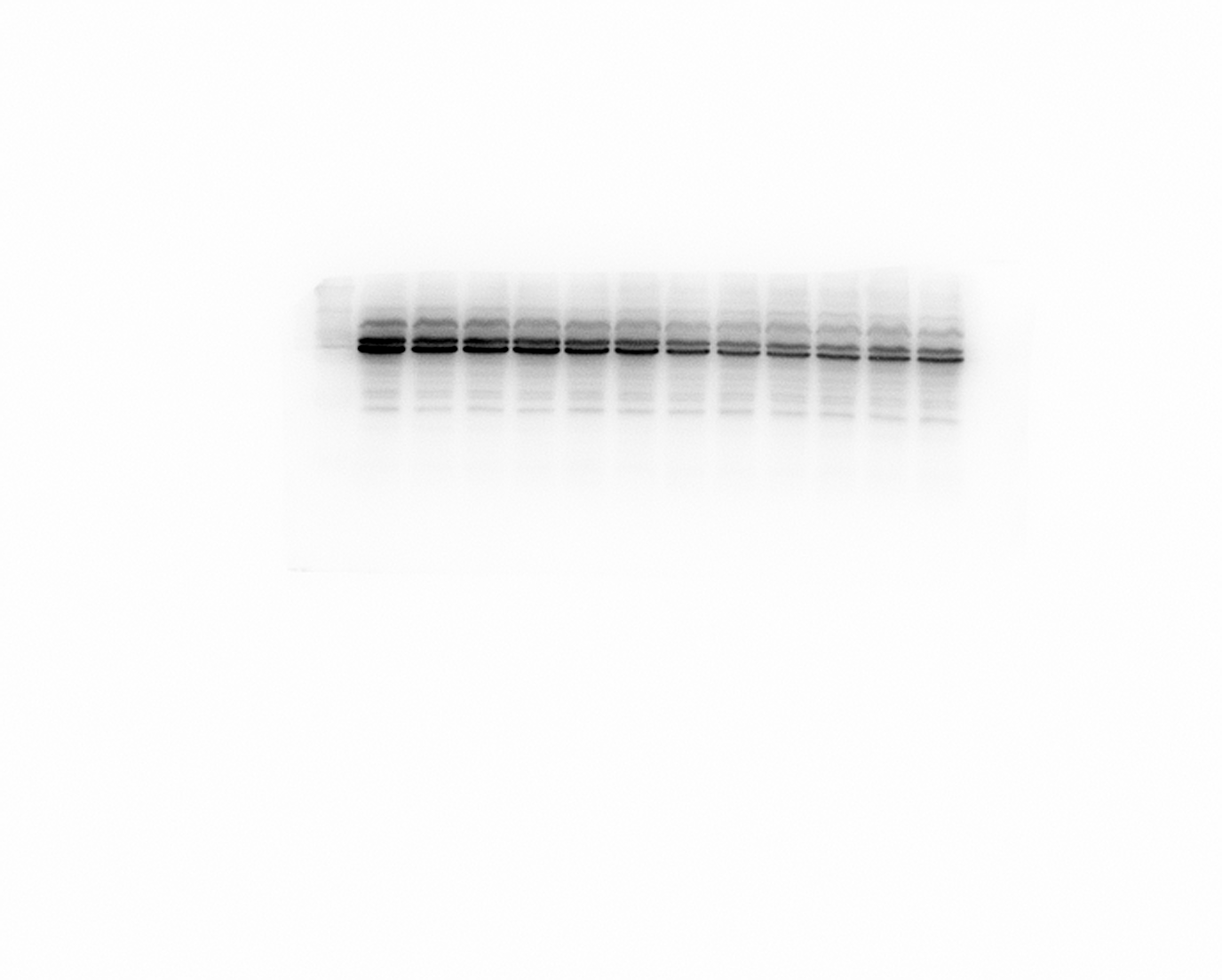

Supplement: Supplementary file 1 [file Data_Sheet_1.ZIP › Supplementary materials 1/WB/ERK.tif]

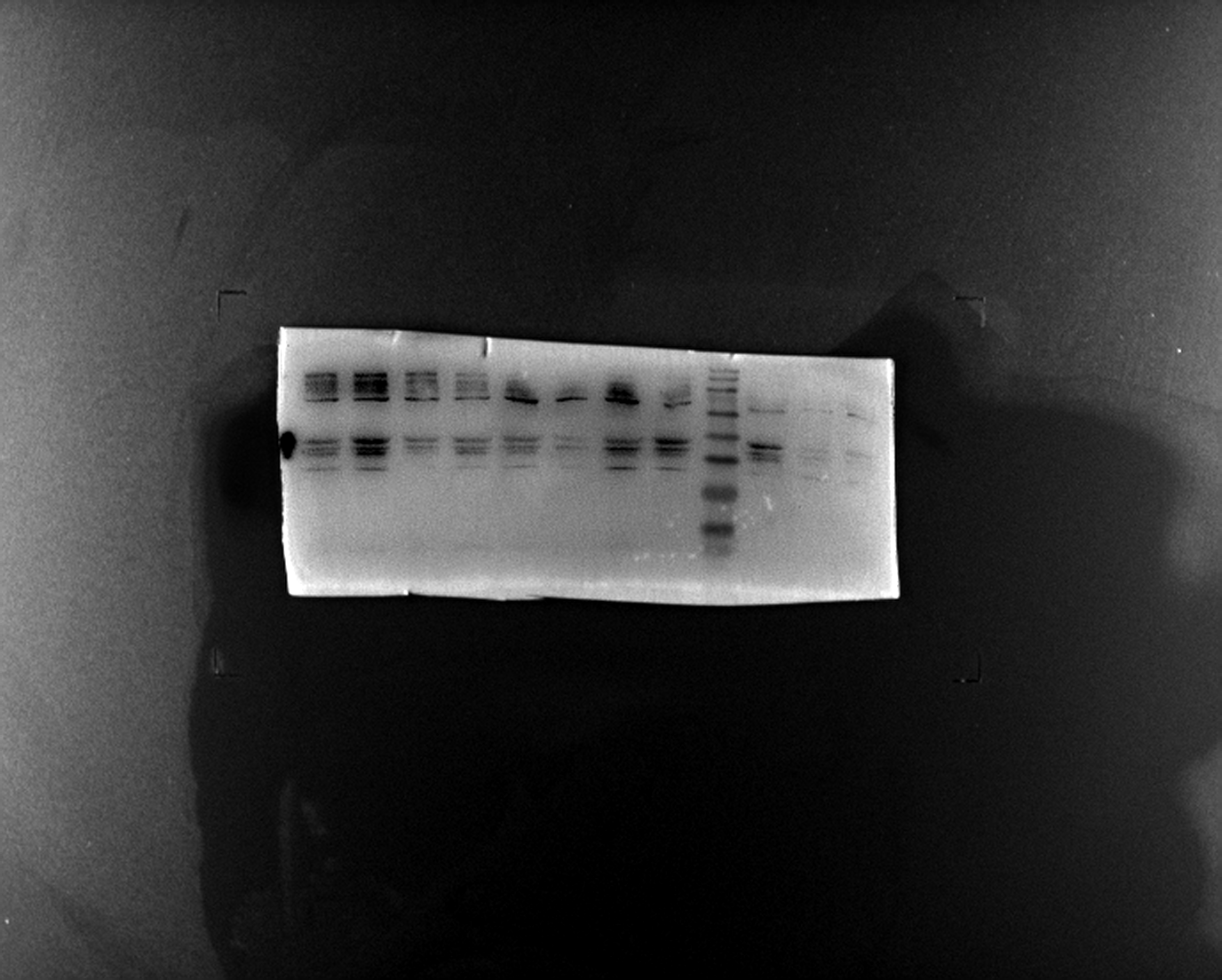

Supplement: Supplementary file 1 [file Data_Sheet_1.ZIP › Supplementary materials 1/WB/p-CREB merge.tif]

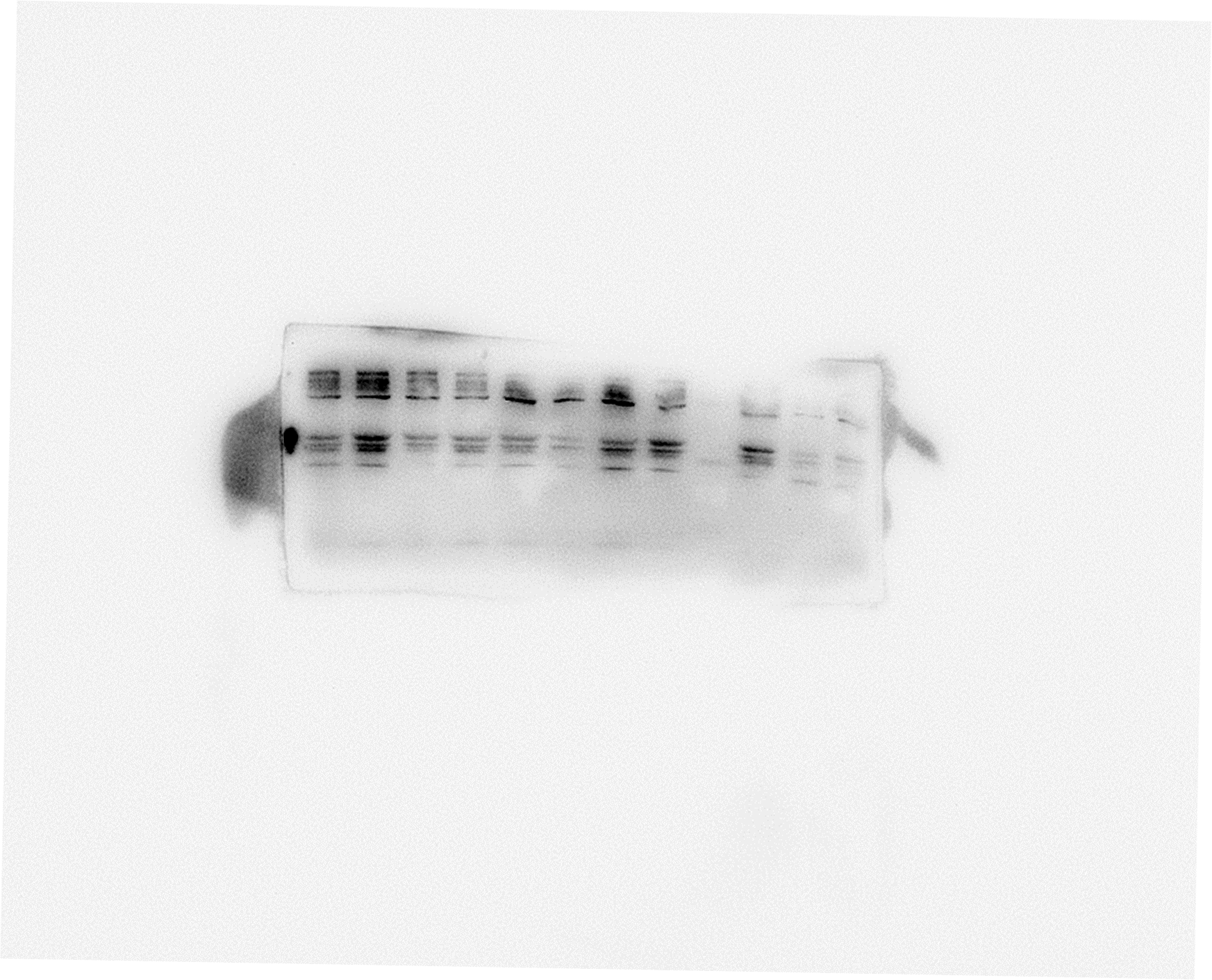

Supplement: Supplementary file 1 [file Data_Sheet_1.ZIP › Supplementary materials 1/WB/p-CREB.tif]

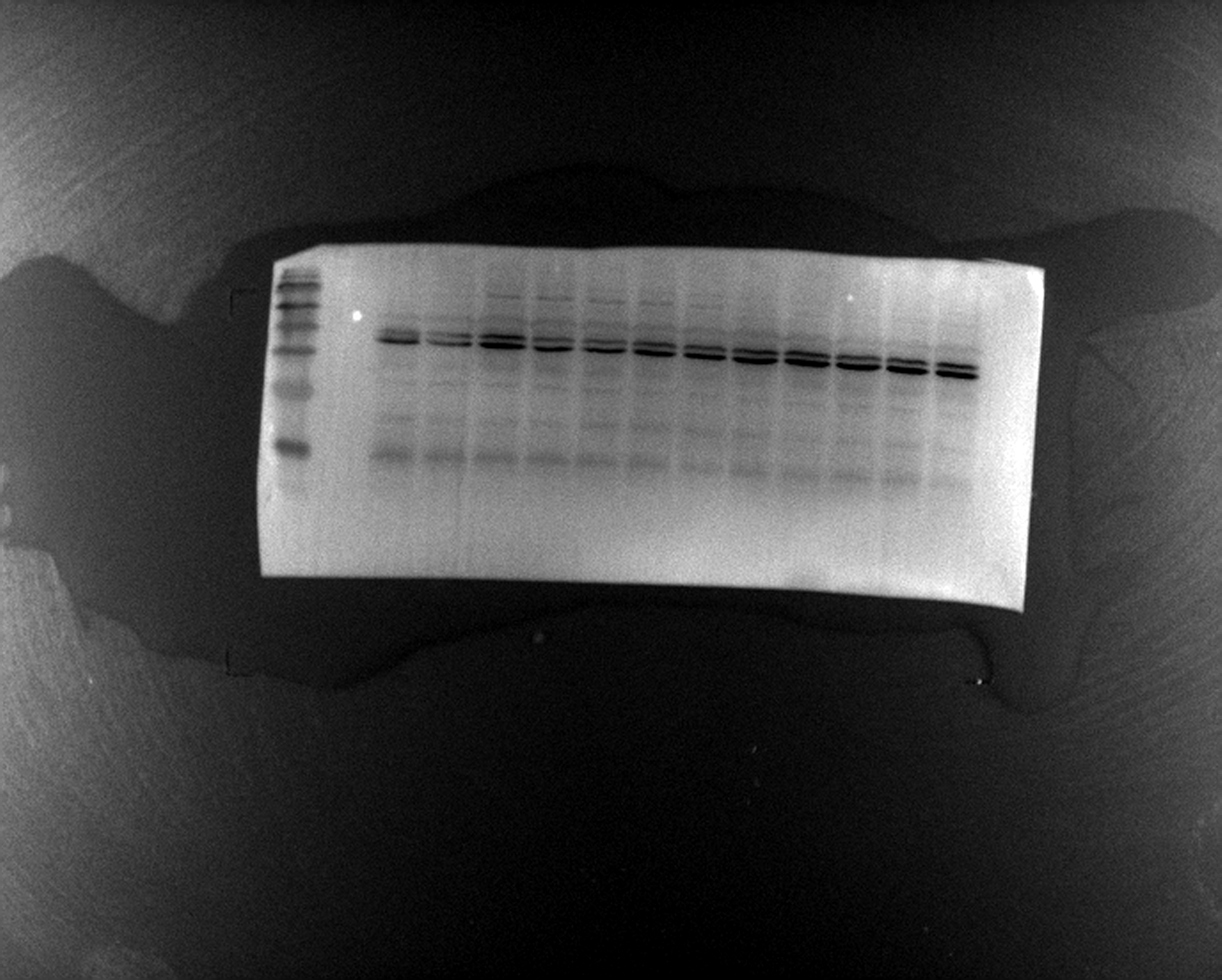

Supplement: Supplementary file 1 [file Data_Sheet_1.ZIP › Supplementary materials 1/WB/p-ERK merge.tif]

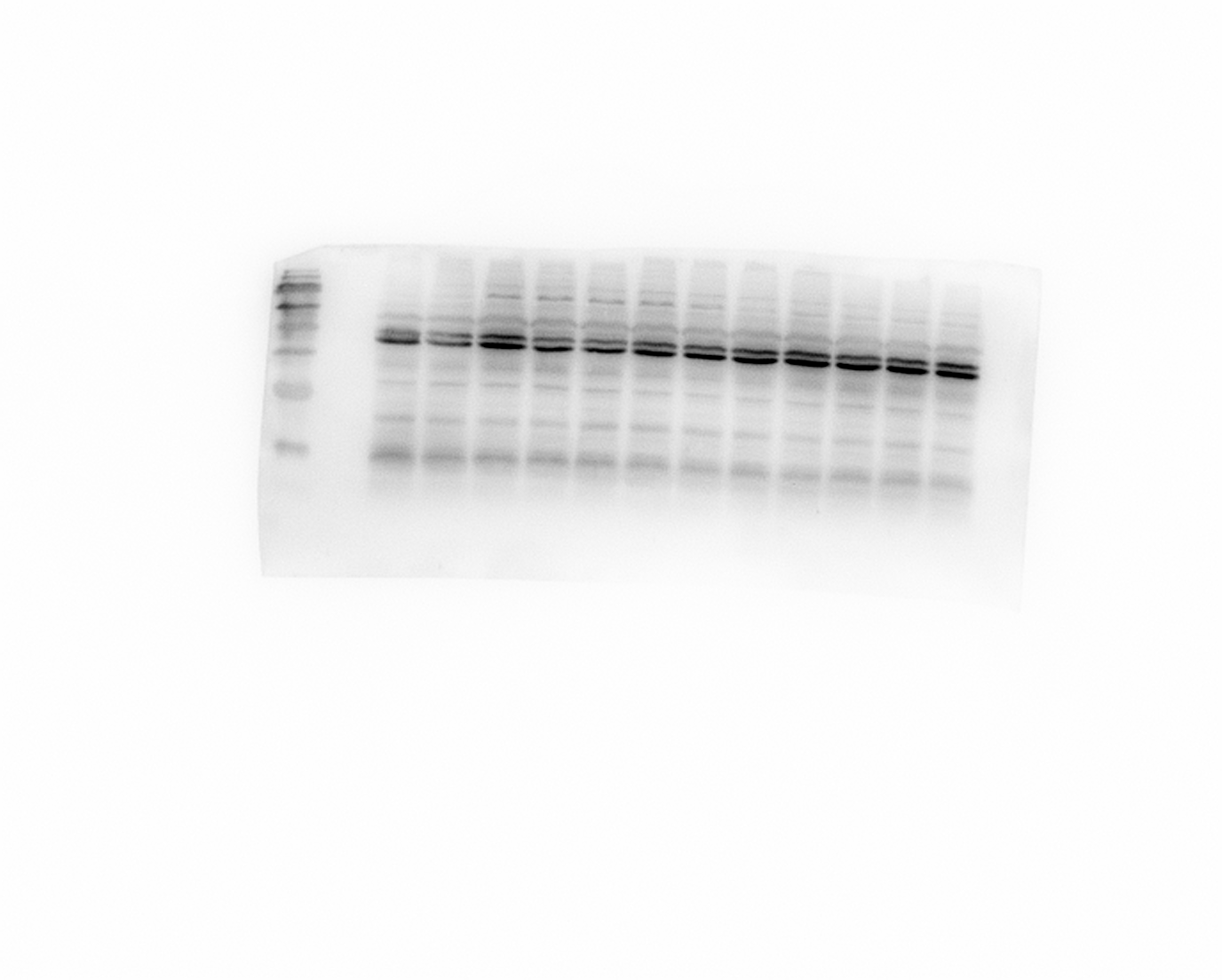

Supplement: Supplementary file 1 [file Data_Sheet_1.ZIP › Supplementary materials 1/WB/p-ERK.tif]

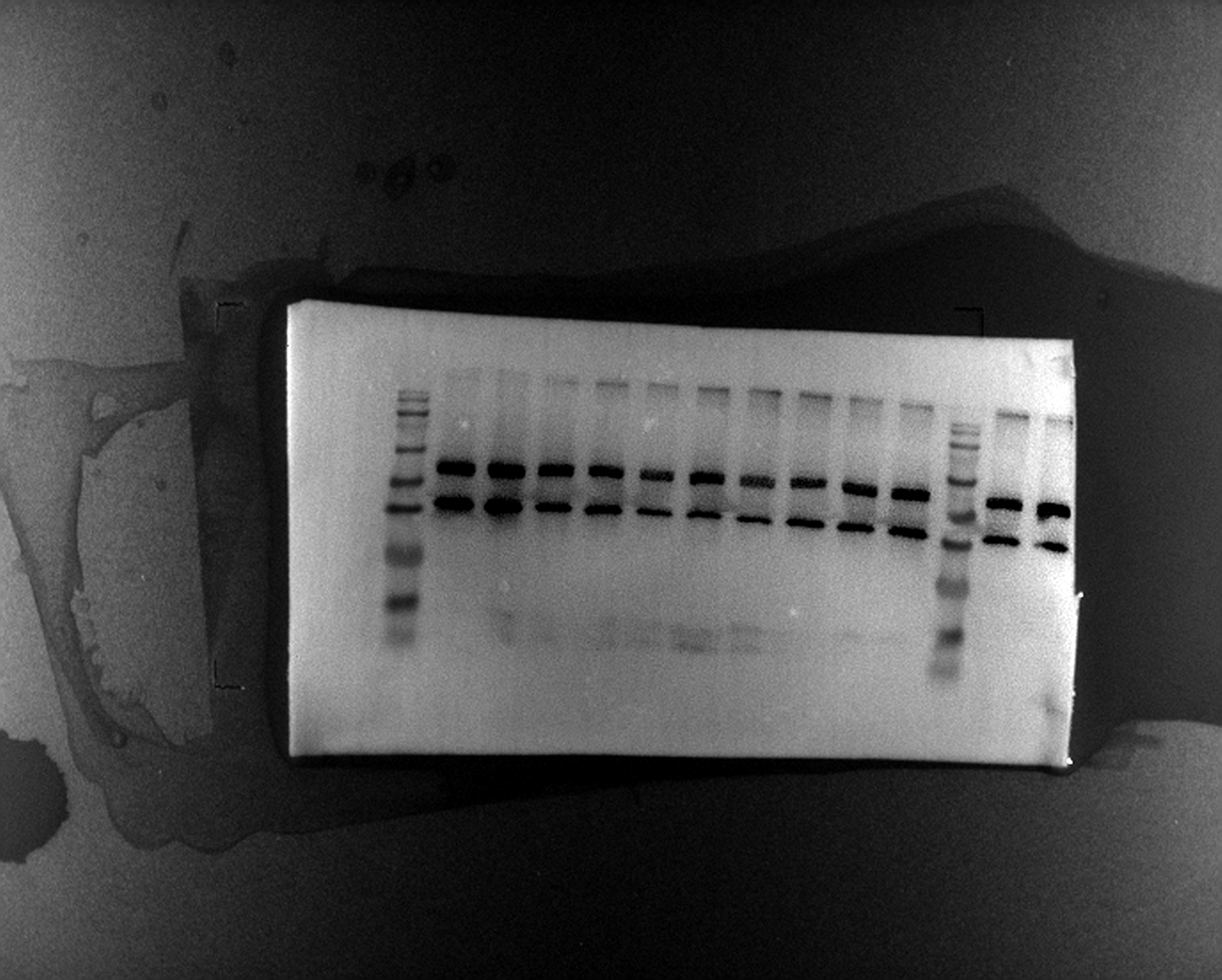

Supplement: Supplementary file 1 [file Data_Sheet_1.ZIP › Supplementary materials 1/WB/PKA MERGE.tif]

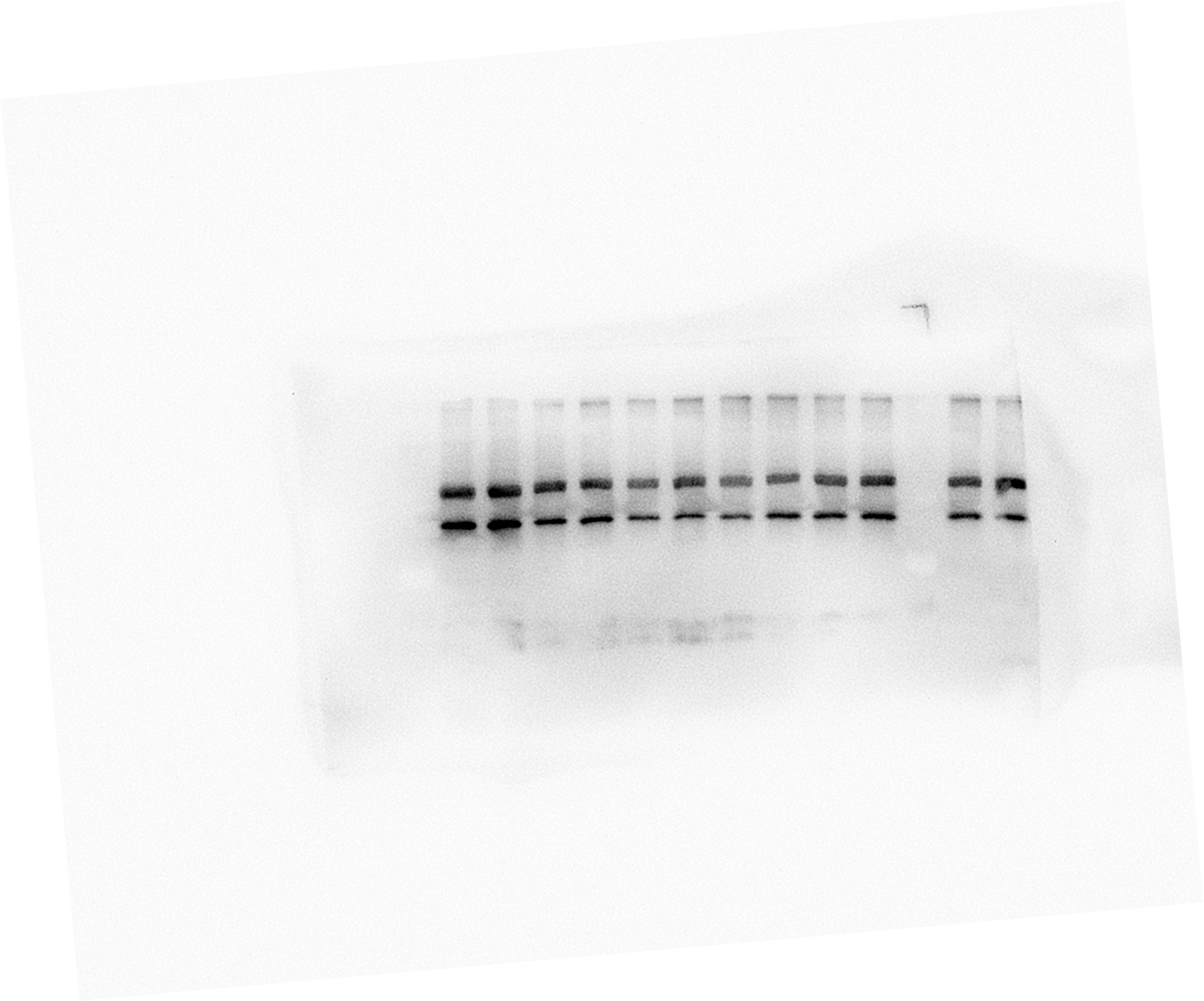

Supplement: Supplementary file 1 [file Data_Sheet_1.ZIP › Supplementary materials 1/WB/PKA.tif]

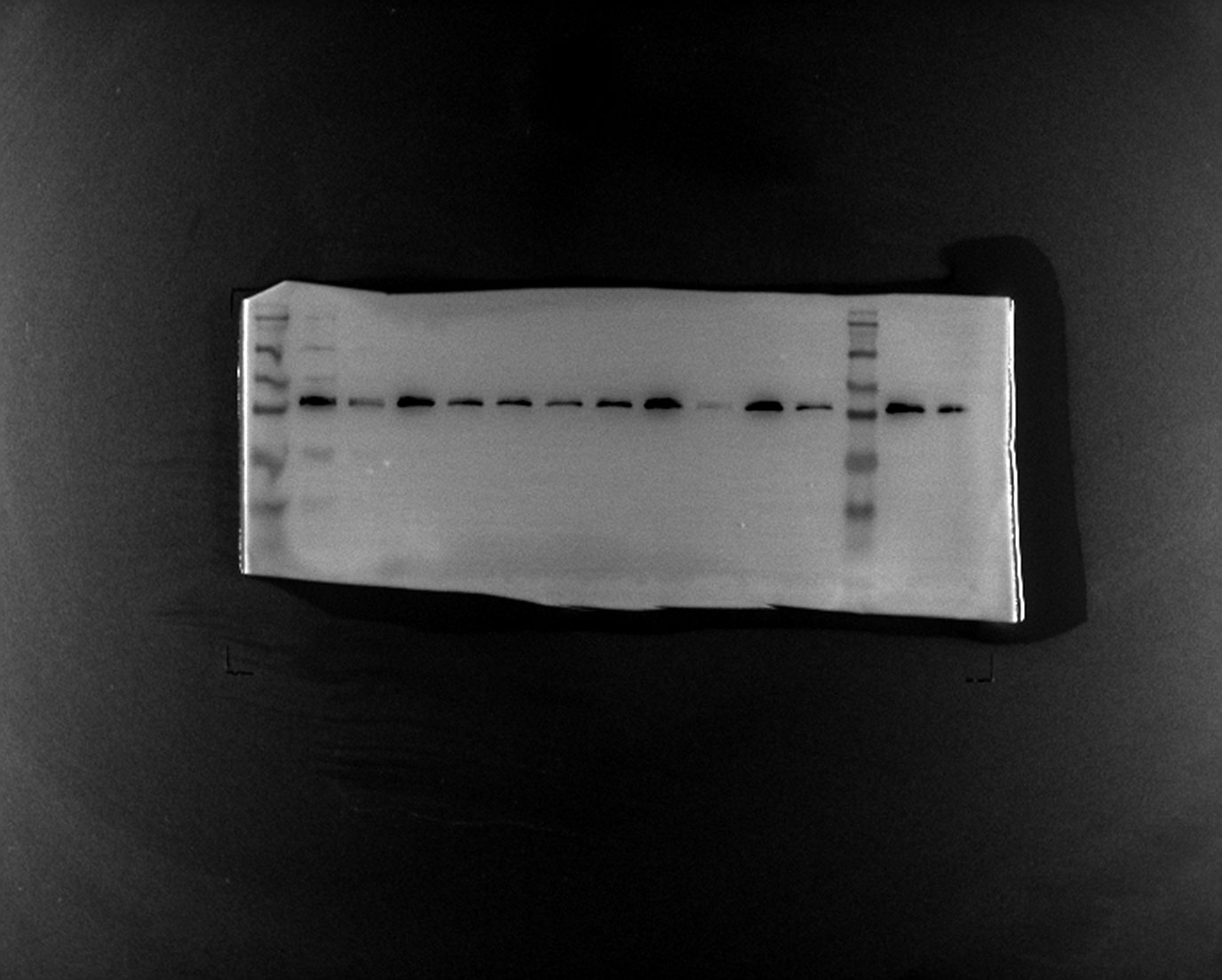

Supplement: Supplementary file 1 [file Data_Sheet_1.ZIP › Supplementary materials 1/WB/p-PKA C MERGE.tif]

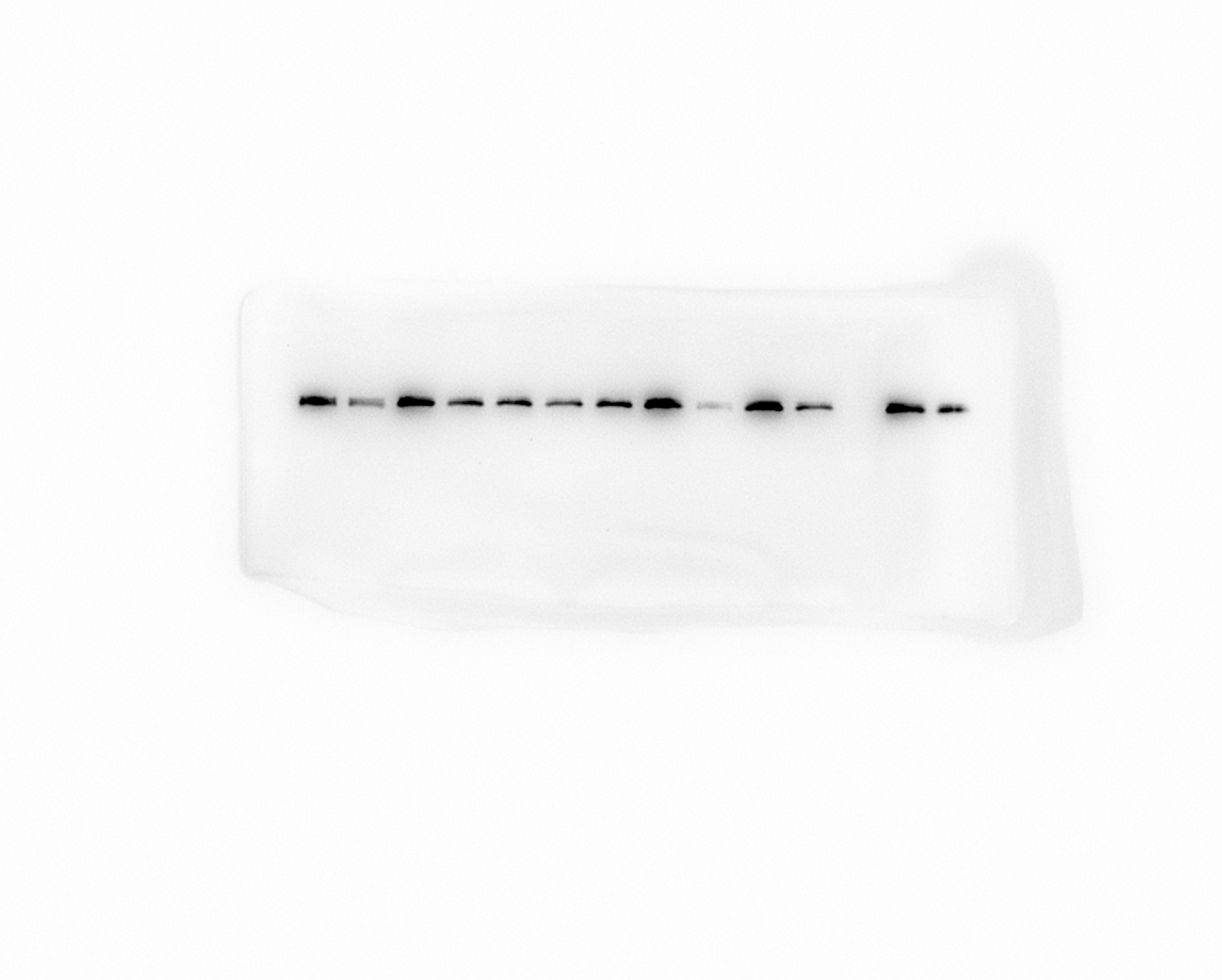

Supplement: Supplementary file 1 [file Data_Sheet_1.ZIP › Supplementary materials 1/WB/p-PKA C.tif]

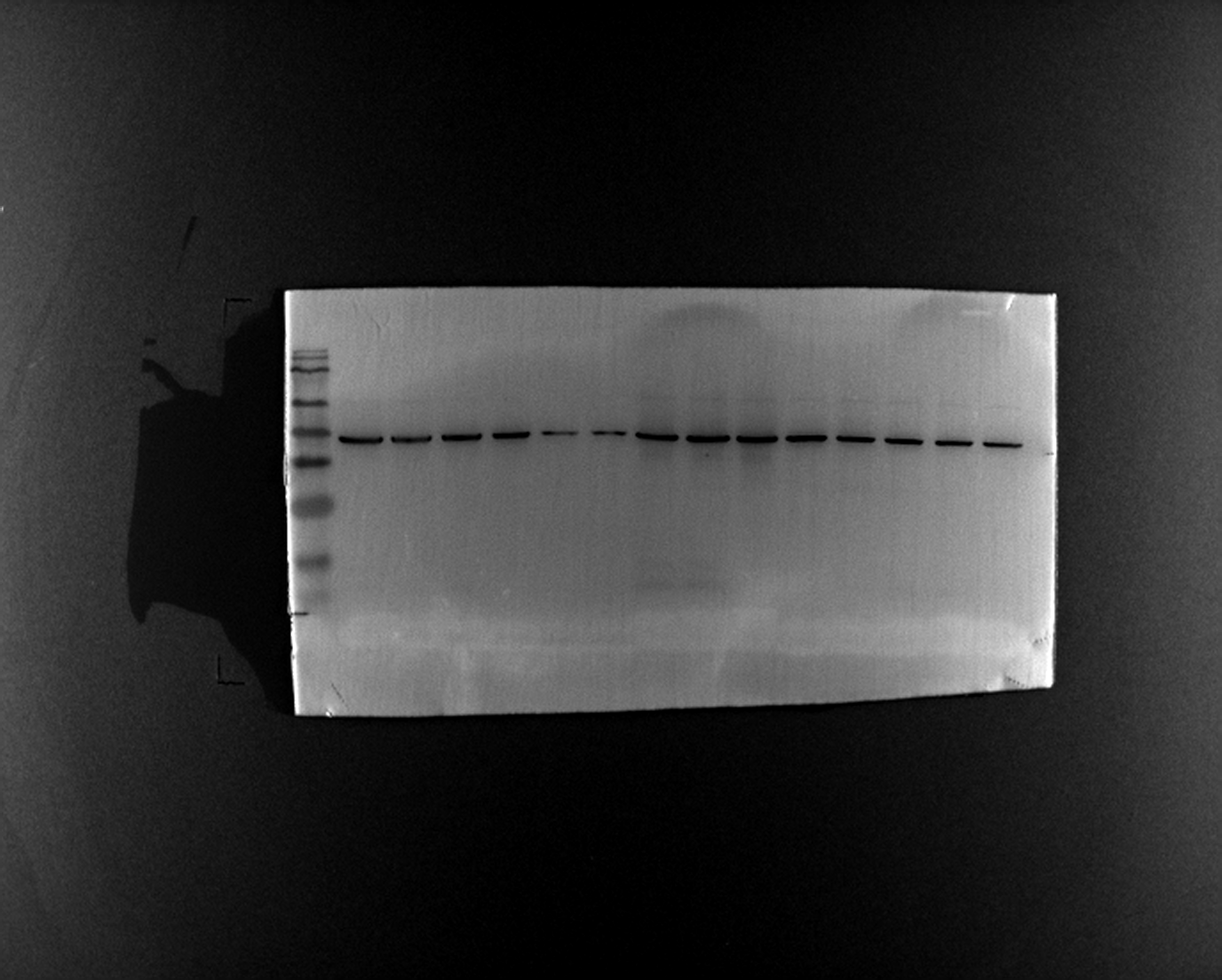

Supplement: Supplementary file 1 [file Data_Sheet_1.ZIP › Supplementary materials 1/WB/a┬-actin merge.tif]

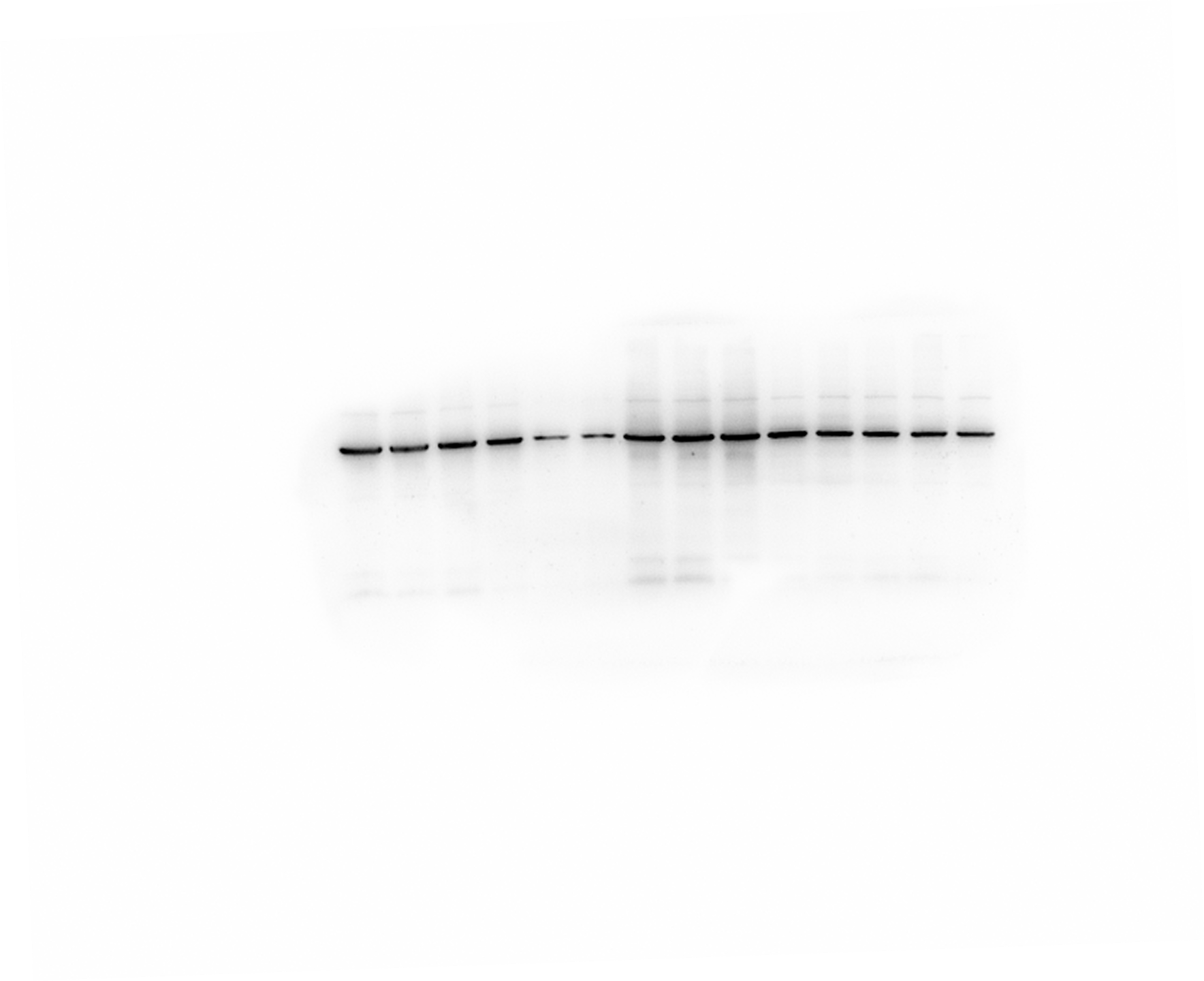

Supplement: Supplementary file 1 [file Data_Sheet_1.ZIP › Supplementary materials 1/WB/a┬-actin.tif]
